# Carotid Intima Media Thickness Reference Intervals for a Healthy Argentinean Population Aged 11–81 Years

**DOI:** 10.1155/2018/8086714

**Published:** 2018-02-14

**Authors:** Alejandro Diaz, Daniel Bia, Yanina Zócalo, Hugo Manterola, Ignacio Larrabide, Lucas Lo Vercio, Mariana Del Fresno, Edmundo Cabrera Fischer

**Affiliations:** ^1^Instituto de Investigación en Ciencias de la Salud, UNICEN, CONICET, Tandil, Argentina; ^2^Physiology Department, School of Medicine, Centro Universitario de Investigación, Innovación y Diagnóstico Arterial (CUiiDARTE), Republic University, General Flores 2125, 11800 Montevideo, Uruguay; ^3^PLADEMA, Facultad de Ciencias Exactas, Universidad Nacional del Centro de la Provincia de Buenos Aires, Tandil, Argentina; ^4^Instituto de Medicina Traslacional, Trasplante y Bioingeniería (IMETTyB), Universidad Favaloro-CONICET, Buenos Aires, Argentina

## Abstract

Reference intervals (RIs) of carotid intima media thickness (CIMT) from large healthy population are still lacking in Latin America. The aim of this study was to determine CIMT RIs in a cohort of 1012 healthy subjects from Argentina. We evaluated if RIs for males and females and for left and right carotids were necessary. Second, mean and standard deviation (SD) age-related equations were obtained for left, right, and average (left + right)/2) CIMT using parametric regression methods based on fractional polynomials, in order to obtain age-specific percentiles curves. Age-specific percentile curves were obtained. Males showed higher A-CIMT (0.577 ± 0.003 mm versus 0.566 ± 0.004 mm, *P* = 0.039) in comparison with females. For males, the equations were as follows: A-CIMT mean = 0.42 + 8.14 × 10^−5^⁎Age^2^; A-CIMT SD = 5.9 × 10^−2^ + 1.09 × 10^−5^⁎Age^2^. For females, they were as follows: A-CIMT mean = 0.40 + 8.20 × 10^−5^⁎Age^2^; A-CIMT SD = 4.67 × 10^−2^ + 1.63 × 10^−5^⁎Age^2^. Our study provides the largest database concerning RIs of CIMT in healthy people in Argentina. Specific RIs and percentiles of CIMT for children, adolescents, and adults are now available according to age and gender, for right and left common carotid arteries.

## 1. Introduction

The early detection of subclinical arterial damage is of value for individual cardiovascular risk assessment and identification of subjects with increased risk (vulnerable subjects) who could benefit from specific preventive strategies [1].

In 1986, common carotid intima media thickness (CIMT) was firstly measured in in vitro studies of arteries from cadaveric donors and noninvasively in ambulatory healthy subjects [2]. Since then, arterial structure characterization through CIMT assessment was introduced in clinical practice and epidemiological investigations demonstrated the prognostic value of this noninvasive study. Common CIMT higher than 0.9 mm has been considered as a factor influencing cardiovascular prognosis by the 2013 ESH/ESC Guidelines for the management of arterial hypertension [3]. Recently, Amato et al. reported that CIMT is an independent predictor of vascular events and should be included in cardiovascular risk models destined to population stratification and preventive strategies [4]. Interestingly, a CIMT score improved the Framingham risk score to predict coronary heart diseases events [4–6]. Also, a recent European consensus reported that increase of CIMT has shown to be marker of hypertension vascular damage and increased cardiovascular risk [5]. Since CIMT is relatively easy to evaluate, the clinical use allows the risk stratification and target organ damage assessment [5, 6].

Age and gender specific percentiles for common CIMT were defined in large populations of healthy subjects and the influence of cardiovascular risk factors (CRFs) was quantified allowing comparative studies among groups with differentiated risk profiles [7]. Epidemiological studies also included pediatric research to characterize age-related CIMT changes in children and adolescent populations for the prediction of cardiovascular events [8].

Ethnicity has demonstrated being an independent predictor of CIMT [9], while intergeographic variations have been observed when comparing data from urban populations from Latin America [10]. Those findings point out that a direct extrapolation and use of CIMT reference intervals (RIs) defined for healthy subjects from European, Asian, or North American populations could not be appropriate and this would be even risky at the time of using CIMT in clinical practice.

Linked to what is mentioned above about factors influencing CIMT, it is noteworthy that left common carotid artery directly originates from the aortic arch, while in the right side the same vessel is a branch of the brachiocephalic trunk. The different origins led to the hypothesis that age, gender, CRFs, and hemodynamic factors would have differential effects on CIMT, depending on the artery analyzed [11]. On the other hand, it has been demonstrated that technical and methodological issues should be considered at the time of CIMT assessment, as well as when analyzing and comparing CIMT data. Then, the development of guidelines and recommendations for standardized CIMT assessment was considered necessary. In this regard, reports from the* Reference Values for Arterial Measurements Collaboration* group (Europe) [7] highlighted the relevance of standardizing both methods and statistical approaches used for vascular evaluation and analysis of large databases.

To the best of our knowledge, there are no works characterizing the CIMT levels and RIs for an urban-rural Argentinean healthy population nonexposed to CRFs, considering a wide age range.

In this context, the purpose of our research was to determine RIs values and age-related CIMT percentile curves in a healthy Argentinean population that included children, adolescents, and adults nonexposed to CRFs. Our data analysis took into account similar methodological considerations to those established by the European group* “Reference Values for Arterial Measurements Collaboration”* [7, 12].

## 2. Materials and Methods

### 2.1. Study Population

This study is part of a project started in 2010 aimed at investigating the prevalence of CRFs. Preliminary data have been previously published [13–16]. The socioeconomic indicators of this population are similar to that of the Argentinean population and other countries of the Southern Cone of Latin America [17, 18].

The protocol of this research was evaluated and approved by the Institutional Ethics and Research Committee. The study was carried on in agreement with the Declaration of Helsinki and the Guidelines for Good Clinical Practice of the European Medicines Agency. Written informed consent was obtained from the participants or those responsible for them.

Asymptomatic subjects from the community were considered for enrollment in this study. Subjects were submitted to clinical interview, blood sampling evaluation, and anthropometric assessment, carried out in all cases by the same group of physicians. Blood samples were obtained after 9–12 hours of fasting. Glycaemia, lipid profile, and kidney functional parameters were determined. Anthropometric evaluation and a brief clinical interview allowed assessing CRFs exposure. Subjects included in the study met the following criteria: (1) normal blood pressure (BP) at the time of examination (being BP < 140/90 mmHg in adults and BP < 90th percentile in younger subjects) [19], (2) no history of cardiovascular, pulmonary, or renal disease; (3) not taking medications (antihyperlipidemic, antihypertensive, or antidiabetic drugs), and (4) all having glycaemia < 6.11 mmol/L (<110 mg/dl), total blood cholesterol levels < 5.17 mmol/L (<200 mg/dl) [3], and normal triglycerides (TG) levels < 1.69 mmol/L (<150 mg/dl) and ≤1.5 mmol/L (<130 mg/dl) for subjects older than 18 years and subjects between 10 to 17 years, respectively [19, 20].

Current and past smokers, diabetic, obese subjects defined by a body mass index (BMI) ≥ 30 kg/m^2^ for adults or BMI ≥ 97th percentile for subjects < 18 years old, hypertensive subjects, or subjects with averaged high BP levels at the time of the study were excluded. To this end, BP measurements were obtained after 5 minutes of sitting rest [3, 19] using fully automatic sphygmomanometers, operating on oscillometric principle (705IT, Omron Healthcare Inc., USA). Adults' BP levels were classified following guidelines for the management of arterial hypertension [3]. Thus, hypertension was defined as systolic BP (SBP) ≥ 140 mmHg and/or diastolic blood pressure (DBP) ≥ 90 mmHg. BP levels in children and adolescents were categorized, considering gender, age, and body height, according to criteria from the American Pediatrics Association and the European Society of Hypertension [19]. Subjects with atherosclerotic plaques in common, internal and/or external carotids, identified during the ultrasonographic study (see below) were excluded.

Based on inclusion and exclusion criteria we defined a population that included 1012 subjects (age range: 11–81 years, males: 61.3%) used to define CIMT RIs (Table 1).

### 2.2. CIMT Measurements

All examinations were performed by a single physician with certified skills in duplex scan diagnostic procedures. All measurements were done in a quiet room with stable temperature (22 ± 1°C) with the patient in supine position, after at least 10 minutes of rest. Studies were done using an Esaote MyLab 40 ultrasound system (Esaote, Genoa, Italy), using a 4–13 MHz linear transducer (LA523).

Left and right CCA and internal and external carotid arteries were scanned and analyzed to verify normal blood flow patterns. Next, sequences of images (videos including at least 5 beats cine-loops), obtained from CCA longitudinal views, were obtained, together with the ECG signal, and stored for offline analysis. Images were obtained in apnea and without swallowing movements. Using specific semiautomatic border detection software (CIMT-tool, Buenos Aires, Argentina), far wall CIMT was measured at end of diastole (peak R wave) selecting the best end diastolic frame out of the loop in the centimeter proximal to carotid bifurcation [21, 22]. CIMT was quantified for right and left common carotid arteries (R-CIMT and L-CIMT, resp.). Additionally, we calculated the averaged CIMT (A-CIMT) as follows: A-CIMT = (R-CIMT + L-CIMT)/2. Readers who did CIMT offline measurements were blinded to participants' identity, age, and sex [10].

### 2.3. Data Analysis

Continuous and categorical data are expressed as mean value ± standard deviation (SD) or percentage, respectively. Data analysis was done using MedCalc Statistical Software (version 14.8.1., MedCalc Inc., Ostend, Belgium) and IBM SPSS 20.0 Software (SPSS Inc., Illinois, USA). A *P* < 0.05 was considered statistically significant.

A stepwise data analysis was done.

*First*, aiming at determining if RIs for R-CIMT and L-CIMT were necessary, we analyzed the degree of equivalence (agreement) between R-CIMT and L-CIMT data by assessing (potential) mean and/or proportional differences (errors) between data and constructing limits of agreement (correlation and Bland-Altman analysis). As a result, specific RIs for R-CIMT and L-CIMT were defined as necessary (Table 2). RIs for A-CIMT values were also defined, allowing analyzing our findings taking into account data from other groups [10, 21].

*Second*, we evaluated whether RIs for males and females were necessary. To this end, bivariate simple and point-biserial correlations between CIMT and subjects' demographic, anthropometric, and blood characteristics were analyzed (Table 3). That analysis enabled identifying variables that should be considered as cofactors in covariate analysis (ANCOVA). Then, sex influence was examined before and after adjustment for cofactors (i.e., age, BP, and total cholesterol) (Table 4). As a result, specific CIMT RIs for males and females were considered necessary (Table 4).

*Third*, age-specific mean and SD equations (for males and females) were obtained for L-CIMT, R-CIMT, and A-CIMT. To this end, parametric regression methods based on fractional polynomials (FPs), as described by Royston and Wright [23], were implemented in MedCalc Software (MedCalc, Ostend, Belgium). Briefly, fitting FPs for age-specific CIMT (right, left, and average) and SD regression curves were defined using iterative procedure (generalized least squares, GLS). The obtained results enabled estimating age-specific mean and SD for CIMT. For instance, CIMT = *a* + *b∗*age^*p*^ + *c∗*age^*q*^ + ⋯, where *a*, *b*, *c*,… are the coefficients and *p*, *q*,… are the powers, with numbers selected from the set [−2, −1, −0.5,0, 0.5,1, 2,3] estimated from the regression for the mean CIMT curve and likewise from the regression for the SD CIMT curve. Continuing the example, FPs with powers [1,2], that is, with *p* = 1 and *q* = 2, illustrate an equation with the form *a* + *b∗*age + *c∗*age^2^ [23]. The residuals were used to assess the model fit, which was deemed appropriate if the residuals were normally distributed, with a mean of 0 and a SD of 1, randomly scattered above and below 0 when plotted against age. The best fitted curves, considering visual and mathematical criteria (Kurtosis and Skewness coefficients), were selected. Then, using the equations obtained for mean and SD, age-specific percentiles were defined using the standard normal distribution (*Z*) (Tables 5, 6, and 7). Age-specific 1st, 2.5th, 5th, 10th, 25th, 50th, 75th, 90th, 95th, 97.5th, and 99th percentile curves were calculated as mean CIMT + *Zp∗*SD, where* Zp* assumed the values of −2.3263, −1.9599, −1.6448, −1.2815, −0.6755, 0, 0.6755, 1.2815, 1.6448, 1.9599, and 2.3263, respectively. The obtained equations were as follows.

(i) For males,

(1) A-CIMT  Mean=0.415946945+0.000081403∗Age2,A-CIMT  SD=0.059010065+0.000010936∗Age2,L-CIMT  Mean=0.45081531+0.000078505∗Age2,L-CIMT  SD=0.056001529+0.000024467∗Age2,R-CIMT  Mean=0.442337809+0.000066517∗Age2,R-CIMT  SD=0.069658789+0.000015017∗Age2.

(ii) For females,

(2) A-CIMT  Mean=0.404023579+0.000082031∗Age2,A-CIMT  SD=0.046702325+0.000016293∗Age2,L-CIMT  Mean=0.404650458+0.000093708∗Age2,L-CIMT  SD=0.054233452+0.00002113∗Age2,R-CIMT  Mean=0.403946979+0.000083633∗Age2,R-CIMT  SD=0.046333378+0.000020631∗Age2.

In the equations, CIMT and age were always expressed in mm and years, respectively.

Considering an 80% and a 95% reference limit and confidence interval (two-sided), respectively, and a 95% and 10% reference range and relative margin of error, respectively, and considering an equally distributed (in three groups at the midpoint and extreme ranges) covariate (age) in the sample, the minimum required sample size was 136 subjects [24].

Finally, the mathematical difference between specific percentiles (75th or 90th) obtained for L-CIMT and R-CIMT was quantified (i.e., 75th percentile for L-CIMT minus 75th percentile for R-CIMT) and analyzed according to age and sex. The analysis (graphic) allowed visualizing how the differences between similar percentiles obtained for left and right CCA were modified with aging, for both males and females.

## 3. Results

### 3.1. General Characteristics of the Analyzed Population

**Figure 1:**
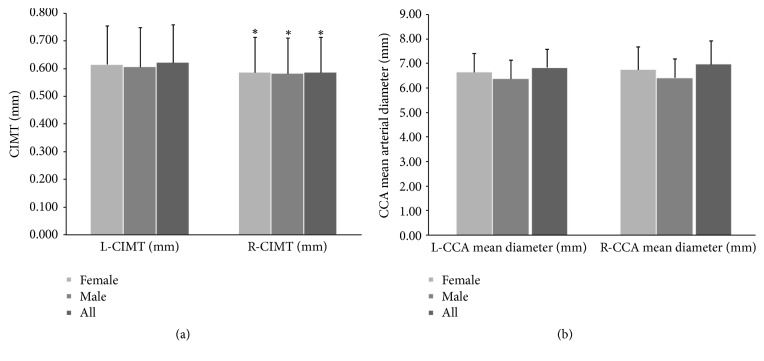
Left and right CIMT (a) and carotid diameter (b) in males, females, and all subject. CIMT: carotid intima media thickness. ^*∗*^Statistically significant difference.

One thousand and twelve healthy subjects were included. Table 1 summarizes data from the entire population and shows findings for males and females. Compared to males, females were (slightly) older and showed lower weight, height, BMI, SBP, MAP, and PP levels (*P* < 0.05) (Table 1). L-CIMT values were higher (*P* < 0.05) than those obtained for R-CIMT. Those differences were observed in the entire population, in males and in females (Figure 1).

A-CIMT value for the entire group was 0.573 ± 0.88 mm, being slightly higher in females (PNS).

### 3.2. CIMT Reference Intervals: Need for Right and Left Specific Determinations

**Figure 2:**
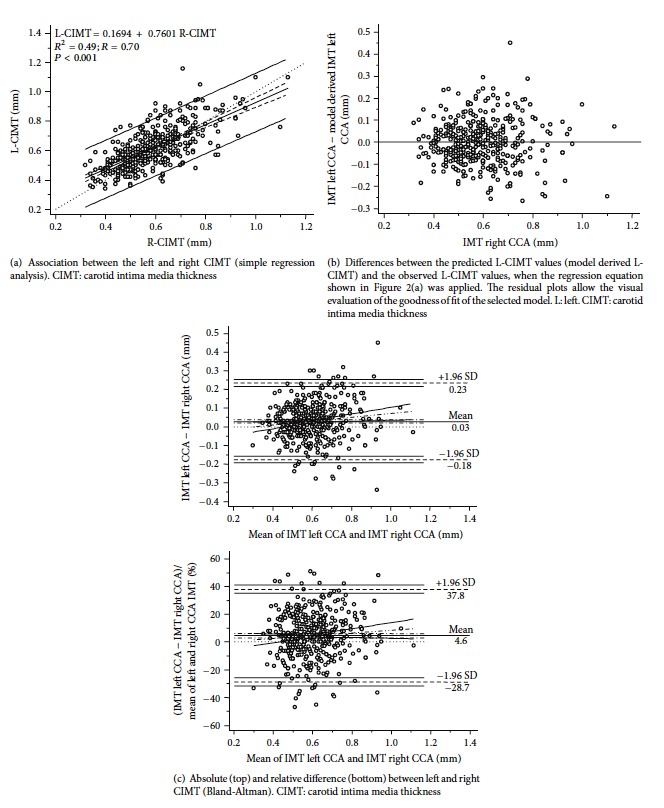


Table 2 and Figure 2(a) show L-CIMT and R-CIMT correlation and the Bland and Altman analysis done to identify potential differences between measurements (Figure 2(b)). As was expected, both determinations showed a significant positive correlation (*P* < 0.0001). In addition, in absolute and relative (percentage) terms, L-CIMT values were higher than R-CIMT (mean or systematic error = 0.029 mm or 4.64%, *P* < 0.0001) (see Figure 2(c)).

There was a proportional error (net slope = 0.097; *P* = 0.0209) between left and right CIMT values. The mentioned differences support the need for specific RIs in analysis of L-CIMT and R-CIMT.

### 3.3. Analysis of CIMT Reference Intervals: Need for Determinations Differentiated by Sex

As can be seen in Table 3, A-CIMT was positively associated with age (*r* = 0.76, *P* = 0.0001), BMI, BP (SBP, DBP, and MAP), glycaemia, cholesterol, and TG levels. It is noteworthy that there were no differences in A-CIMT between males and females (Table 3) before adjusting for covariates (Table 4). However, after adjusting (ANCOVA analysis) for age, total cholesterol, TG, and glycaemia, there were sex-related differences in A-CIMT (Table 4). Compared to females, males showed higher A-CIMT values (0.577 ± 0.003 mm versus. 0.566 ± 0.004 mm, *P* = 0.039). The sex-related differences in A-CIMT supported the need for RIs differentiated by gender.

As can be seen in Table 4 differences in A-CIMT between males and females could be explained considering the physiological differences in BMI and/or BP, since when the model was adjusted by those cofactors, the sex-related differences in A-CIMT disappeared.

### 3.4. CIMT Reference Intervals (Percentile Analysis)

Age-specific (5-year intervals, RIs) percentile analyses for A-CIMT corresponding to males and females are shown in Tables 5 and 6, respectively. Similarly, in the Supplementary Materials, Tables A and B (for males and females, resp.) show the RIs for A-CIMT defined for each year of age.

Age-specific (5-year RIs) percentile analyses of R-CIMT corresponding to males and females are shown in Tables 7 and 8, respectively. In the Supplementary Materials, Tables C and D show data for year of age in both males and females, respectively.

The age-specific (5-years RIs) percentile analysis of L-CIMT corresponding to males and females are shown in Tables 9 and 10, respectively. In the Supplementary Materials, Tables E and F, show data for year of age both, in males and females, respectively.

**Figure 3:**
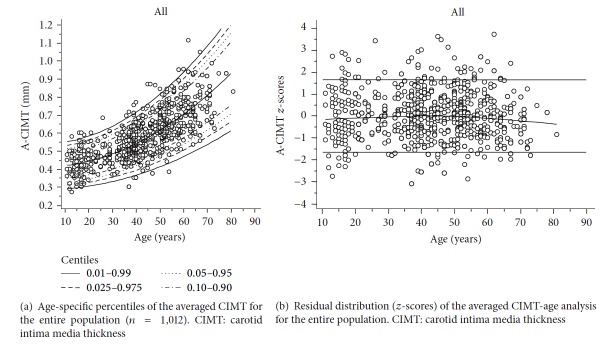


**Figure 4:**
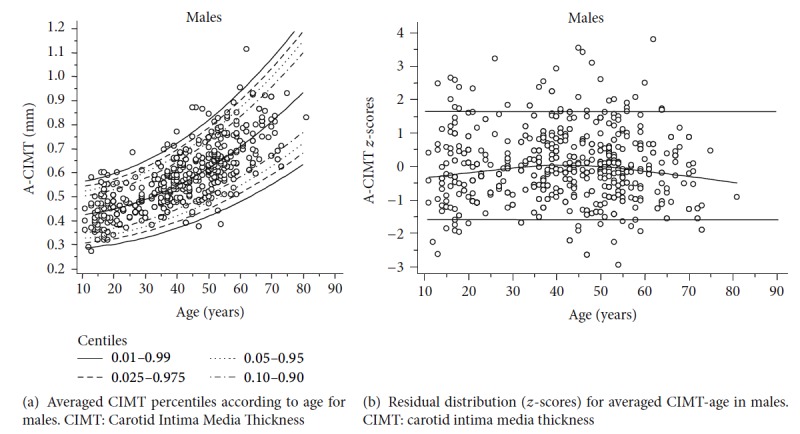


**Figure 5:**
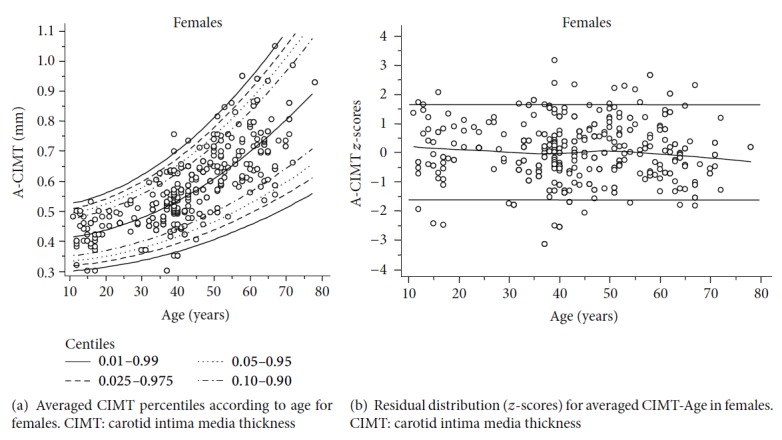


Figures 3, 4, and 5 show age and sex-specific A-CIMT percentile lines superimposed on the raw data (part (a) of each figure) and the residual distribution for A-CIMT values according to age (part (b) of each figure), for the whole population, males and females, respectively.

**Figure 6:**
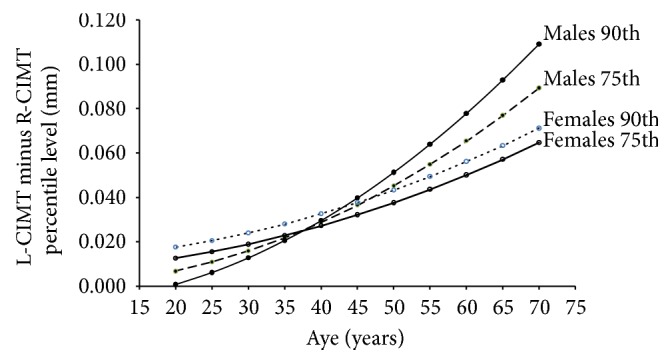
Percentile differences (75th and 90th) between L-CIMT and R-CIMT according to age and sex. L: left. R: right. CIMT: carotid intima media thickness.

Percentile differences (75th and 90th) between L-CIMT and R-CIMT increased with age in both, males and females (Figure 6). The increase was more pronounced in males than in females. However, while percentile 75th and 90th showed an age-related increase in females, in males there was an inverse relationship before and after a given age (between 35 and 40 y.). As can be seen in Figure 6, 90th percentile showed the lowest values in younger males, while in older males exhibiting the highest differences between L-CIMT and R-CIMT.

## 4. Discussion

Most of the studies in which RIs for CIMT were defined included data obtained from retrospective analysis of patients evaluated in different specialized centers [4, 7, 9, 25]. On the other hand, differences in inclusion/exclusion criteria and/or in the methodological approaches make it difficult to carry out comparative analyses among studies and/or to extrapolate data to other populations.

Despite the recognized value of CIMT for predicting cardiovascular risk, in the Southern Cone of Latin America there is a scarcity of RIs. To the best of our knowledge, three research groups published reference values for CIMT in Latina America. CARMELA study reported normal CIMT values obtained from the study of 3071 subjects (25–64 years), from 7 cities of urban Latin America [10]. On the other hand, RIs for CIMT based on urban population screening have only been reported from Uruguay [26] and Peru [27].

In this context, the present research shows RIs for CIMT with respect to age, providing relevant clinical information in terms of carotid wall structure. About this, changes in CIMT have been described in association with aging, which should be taken into account when using CIMT in clinical practice. About this, the clinician should know expected mean and deviations values, so as to adequately interpret CIMT data obtained in a given subject and to orientate both diagnosis and preventive strategies.

Our work has methodological strengths and relevant findings that should be remarked.

*First,* this work represents the first rural-urban Argentine population based study aiming at determining CIMT reference values in a large number of normotensive and healthy subjects nonexposed to CRFs. It should be noted that demographic and sociocultural characteristics of the population from Tandil have similarities with the other populations from Argentina and South America [17]. About this, adequate interpretation and application of data obtained in this work require taking into account the studied population context and characteristics. In turn and in agreement with what is stated above, data could only be extrapolated to those communities with similar characteristics.

*Second,* the number of subjects included in our research is similar to that mentioned in the specialized literature (international databases) [28, 29], but in this work we considered a wider age range (11 to 81 years). CARMELA study included 3071 adults and healthy subjects between 25 and 64 years [10]. Therefore, no data about RIs for CIMT in the adolescents and elderly subjects were defined in the mentioned work. That would be considered an important issue, since it is known that elderly population would represent up to 15% of the total population of the Southern Cone countries [17]. On the other hand, the number of subjects included in other works is significantly smaller than that of this work. In Latin America Pastorius et al. reported CIMT RIs for adult males and females (20–80 years) based on 472 healthy subjects from Peru [27]. In Uruguay, Farro et al. reported RIs for CIMT in 367 subjects including adolescents selected from an urban population [26]. The European Registry of Reference Values for Arterial Measurements Collaboration reported normal values of CIMT based on 4234 records. Those values were obtained from a retrospective analysis of CIMT assessed with echotracking in 24 European centers of high complexity [7]. Then, the “normal population” was in fact a highly selected group of subjects whose characteristics would be quite different from those observed in a typical patient in daily clinical practice. Related to that, it is noteworthy that the* “normal population”* represented only 16.8% of the studied patients. The CAMP study by Ciccone et al. established that the percentiles for normal CIMT involved 1017 healthy subjects aged between 22 and 85 years from Italian centers. In this study CIMT was positively correlated with age and mean values were higher in men than in women [29]. This interesting report shows results in terms of CIMT RIs similar to those found in our research.

*Third,* we found sex-related differences in CIMT values. That finding is in agreement with data reported by other groups [7, 29]. The European “Reference Values of Arterial Measurements Collaboration Group” reported RIs and percentiles for each age group (from 15 to 85 years) separated by sex [7]. In CARMELA study mean CIMT and IC 95% were reported for males and females, separately [10]. Pastorius et al. reported RIs and specific percentile curve according to age and gender based on 207 males and 252 females [27].

*Fourth*, we found differences in the aging-related increase in CIMT when comparing left and right sides. As can be seen in Figure 6, the differences between right and left CIMT corresponding to percentiles 75th and 90th increased with age in both males and females. However, some issues should be analyzed considering the age-associated increases of the differences between L-CIMT and R-CIMT, which were not uniformly distributed:

(a) The finding of differences between L-CIMT and R-CIMT highlights the need for right and left CMIT evaluation.

(b) Left and right CIMT measurements are particularly necessary in older males, taking into account the fact that the differential behavior is more pronounced in men than in females.

(c) Despite percentiles 75th and 90th showed an age-associated increased in both sexes, in males in early ages (before 35–40 years) the 90th percentile showed the greatest increases.

Jointly considering what is stated above emphasizes the need for specific age-related left and right CIMT RIs for males and females.

### 4.1. Methodological Considerations

Recently, Dalla Pozza et al. commented on the lack of recommendations (universally accepted) on when and how the CIMT should be measured in a particular subject [8]. In addition, the authors emphasized that data analysis should be performed taken into account previous findings using a validated methodology. In this regard, we are convinced that this is an important issue and consequently in this research data analysis applied similar methodological approach to that used by the European group* “Reference Values for Arterial Measurements Collaboration”* [7, 12].

### 4.2. Limitations of This Research

This research used a cross-sectional design. Then, the age-associated increase in CIMT described would not represent aging-related changes in CIMT for a subject. An adequate data analysis, interpretation, and use in clinical practice should take into account what is stated above.

## 5. Conclusions

This study provides the largest database concerning RIs of CIMT in healthy rural-urban people in Argentina. CIMT RIs and percentiles were defined by age and sex considering a healthy population aged 11–81 years. In addition, specific RIs and percentiles of CIMT for right and left common carotid artery were reported.

## Figures and Tables

**Table 1:** Subjects characteristics.

	*All *(*n* = 1012)	*Female *(*n* = 391)	*Males *(*n* = 621)	*P* value
	MV ± SD	MV ± SD	MV ± SD	
Age [years]	42 ± 15	43 ± 14	41 ± 15	0.026
Body weight [Kg.]	66.5 ± 12.0	57.5 ± 11.0	72.5 ± 12.0	<0.001
Body height [cm]	166.0 ± 10.7	160.0 ± 10.0	170.0 ± 11.0	<0.001
BMI [Kg./m^2^]	24.1 ± 3.1	22.6 ± 2.7	25.1 ± 2.9	<0.001
Total cholesterol [mg/dl]	155.5 ± 23.9	155.2 ± 23.9	155.7 ± 24.0	0.765
Triglycerides [mg/dl]	74.0 ± 22.4	73.7 ± 23.1	74.1 ± 21.9	0.789
Glycemia [mg/dl]	82.1 ± 8.8	82.1 ± 8.8	82.1 ± 8.8	0.986
Creatinine [mg/dl]	0.9 ± 2.5	0.8 ± 0.1	1.0 ± 3.1	0.417
Hematocrit [%]	41.7 ± 2.1	41.7 ± 2.1	41.7 ± 2.1	0.824
SBP [mmHg]	119 ± 11	115 ± 12	122 ± 10	<0.001
MBP [mmHg]	92 ± 9	89 ± 10	93 ± 8	<0.001
DBP [mmHg]	73 ± 8	72 ± 9	74 ± 8	0.002
PP [mmHg]	46 ± 8	43 ± 8	48 ± 7	<0.001
HR [beats/minute]	65 ± 10	66 ± 10	65 ± 9	0.205
A-CIMT [mm]	0.573 ± 0.885	0.574 ± 0.132	0.572 ± 0.128	0.753
A-CCA mean diameter [mm]	6.45 ± 0.88	6.26 ± 0.89	6.57 ± 0.86	<0.001

MV: mean value. SD: standard deviation. SBP, MBP, DBP, and PP: systolic pulse pressure, mean pulse pressure, diastolic pulse pressure, and pulse pressure. HR: heart rate. A-CIMT: common carotid artery intima media thickness average value. A-CCA: common carotid artery diameter average value. Average values are the mean of left and right measurements. Statistics: *t*-test (unpaired, two-tailed) analyzed differences between males and females. *P* < 0.05 was considered statistically significant.

**Table:** (a) Regression analysis (left CIMT: *y*-axis, right CIMT: *x*-axis)

*R* ^2^	0.4856
Regression equation	*y* = 0.1694 + 0.7601*x*
Intercept	
Coefficient	0.1694
SE	0.02339
95% CI	0.1234 to 0.2154
*P*	<0.0001
Slope	
Coefficient	0.7601
SE	0.03902
95% CI	0.6834 to 0.8368
*P*	<0.0001

SE: standard error. CI: confidence interval.

**Table:** (b) Differences (Bland & Altman)

	Left CIMT − right CIMT [mm]	Left − right CIMT/mean of left and right CIMT [%]
Arithmetic mean	0.029	4.641
95% CI	0.01899 to 0.03933	2.9958 to 6.2852
*P* (H_0_: mean = 0)	<0.0001	<0.0001
SD	0.104	16.816
Lower limit	−0.175	−28.319
95% CI	−0.1920 to −0.1572	−31.1321 to −25.5058
Upper limit	0.233	37.600
95% CI	0.2156 to 0.2503	34.7867 to 40.4130

Regression equation	*y* = −0.02913 + 0.09710*x*	*y* = −0.5378 + 8.6262*x*

Intercept		
Coefficient	−0.029	−0.538
SE	0.026	4.170
*t*-value	−1.135	−0.129
*P*	0.257	0.897
95% CI	−0.07958 to 0.02132	−8.7351 to 7.6595

Slope		
Coefficient	0.097	8.626
SE	0.042	6.805
*t*-value	2.318	1.268
*P*	0.0209	0.206
95% CI	0.01476 to 0.1794	−4.7520 to 22.0044

SE and SD: standard error and standard deviation. Ho: null hypothesis. CI: confidence interval.

**Table 3:** Correlations between A-CIMT and subjects demographic, anthropometric, and blood characteristics.

	A-CIMT [mm]
Age [years]	
*R*	0.76
*P* value	**0.00**
Sex (1: male; 0: female)	
*R*	−0.01
*P* value	**0.75**
BMI [Kg./m^2^]	
*R*	0.32
*P* value	**0.00**
SBP [mmHg]	
*R*	0.23
*P* value	**0.00**
DBP [mmHg]	
*R*	0.39
*P* value	**0.00**
MBP [mmHg]	
*R*	0.34
*P* value	**0.00**
Glycemia [mg/dl]	
*R*	0.06
*P* value	**0.05**
Total cholesterol [mg/dl]	
*R*	−0.13
*P* value	**0.00**
Triglycerides [mg/dl]	
*R*	0.07
*P* value	**0.02**

A-CIMT: average between left and right common carotid intima media thickness values. SBP, MBP, DBP, and PP: systolic pulse pressure, mean pulse pressure, diastolic pulse pressure, and pulse pressure. *P* < 0.05 was considered statistically significant.

**Table 4:** Sex-related common carotid intima media thickness (A-CIMT) comparison: analysis of covariance (ANCOVA) adjusting by age and other cardiovascular risk factors.

	*CIMT before adjustment*		*CIMT after adjustment*				*Covariates appearing in the model are evaluated at the*						
							*following values*						
	MV ± SE	*P* value	MV ± SE	*P* value	95% CI Lower limit	95% CI Upper limit	Age [years]	Total cholesterol [mg/dl]	Glycemia [mg/dl]	Triglycerides [mg/dl]	BMI [Kg./m^2^]	SBP [mmHg]	DBP [mmHg]
Male	0.572 ± 0.128	0.753	0.577 ± 0.003	0.037	0.570	0.584	**41.91**	-* *-* *-* *-* *-	-* *-* *-* *-* *-	-* *-* *-* *-* *-	-* *-* *-* *-* *-	-* *-* *-* *-* *-	-* *-* *-* *-* *-
Female	0.574 ± 0.132		0.566 ± 0.004		0.557	0.574							
Male	0.572 ± 0.128	0.753	0.577 ± 0.003	0.039	0.570	0.584	**41.91**	**155.49**	**82.131**	**73.96**	-* *-* *-* *-* *-	-* *-* *-* *-* *-	-* *-* *-* *-* *-
Female	0.574 ± 0.132		0.566 ± 0.004		0.557	0.574							
Male	0.572 ± 0.128	0.753	0.575 ± 0.004	0.322	0.568	0.582	**41.91**	-* *-* *-* *-* *-	-* *-* *-* *-* *-	-* *-* *-* *-* *-	**24.1218**	-* *-* *-* *-* *-	-* *-* *-* *-* *-
Female	0.574 ± 0.132		0.569 ± 0.005		0.560	0.578							
Male	0.572 ± 0.128	0.753	0.570 ± 0.003	0.402	0.568	0.581	**41.91**	-* *-* *-* *-* *-	-* *-* *-* *-* *-	-* *-* *-* *-* *-	-* *-* *-* *-* *-	**119.23**	**73.21**
Female	0.574 ± 0.132		0.575 ± 0.004		0.561	0.578							

A-CIMT: average between left and right common carotid intima media thickness values. BMI: body mass index. SBP and DBP: systolic blood pressure and diastolic blood pressure. MV: mean value. SD: standard deviation. SE: standard error. CI: confidence interval. *P* < 0.05 was considered statistically significant.

**Table 5:** Common carotid intima media thickness [mm] percentiles for healthy male subjects (average of right and left arterial measurements).

Age [years]	1st	2.5th	5th	10th	25th	50th	75th	90th	95th	97.5th	99th
15	0.2913	0.3138	0.3332	0.3555	0.3927	0.4343	0.4758	0.5130	0.5354	0.5547	0.5773
20	0.3011	0.3243	0.3442	0.3673	0.4057	0.4485	0.4913	0.5297	0.5528	0.5727	0.5960
25	0.3136	0.3378	0.3585	0.3824	0.4223	0.4668	0.5113	0.5512	0.5751	0.5959	0.6200
30	0.3290	0.3543	0.3760	0.4010	0.4427	0.4892	0.5357	0.5774	0.6025	0.6242	0.6494
35	0.3472	0.3738	0.3966	0.4229	0.4668	0.5157	0.5646	0.6085	0.6348	0.6576	0.6841
40	0.3682	0.3962	0.4203	0.4481	0.4945	0.5462	0.5979	0.6442	0.6720	0.6961	0.7242
45	0.3920	0.4217	0.4473	0.4768	0.5260	0.5808	0.6356	0.6848	0.7143	0.7398	0.7696
50	0.4186	0.4502	0.4774	0.5088	0.5611	0.6195	0.6778	0.7301	0.7615	0.7887	0.8203
55	0.4480	0.4817	0.5107	0.5442	0.6000	0.6622	0.7244	0.7802	0.8137	0.8427	0.8764
60	0.4801	0.5162	0.5472	0.5829	0.6425	0.7090	0.7755	0.8351	0.8708	0.9018	0.9379
65	0.5151	0.5537	0.5868	0.6250	0.6888	0.7599	0.8309	0.8947	0.9329	0.9661	1.0046
70	0.5529	0.5941	0.6296	0.6705	0.7388	0.8148	0.8909	0.9591	1.0000	1.0355	1.0768
75	0.5935	0.6376	0.6756	0.7194	0.7924	0.8738	0.9553	1.0283	1.0721	1.1101	1.1542
80	0.6368	0.6841	0.7247	0.7716	0.8498	0.9369	1.0241	1.1022	1.1491	1.1898	1.2370

**Table 6:** Common carotid intima media thickness [mm] percentiles for healthy female subjects (average of right and left arterial measurements).

Age [years]	1st	2.5th	5th	10th	25th	50th	75th	90th	95th	97.5th	99th
15	0.3053	0.3238	0.3396	0.3579	0.3885	0.4225	0.4565	0.4870	0.5053	0.5212	0.5397
20	0.3130	0.3325	0.3493	0.3686	0.4009	0.4368	0.4728	0.5050	0.5244	0.5411	0.5606
25	0.3230	0.3438	0.3617	0.3824	0.4169	0.4553	0.4937	0.5282	0.5489	0.5668	0.5876
30	0.3351	0.3576	0.3769	0.3992	0.4364	0.4779	0.5193	0.5565	0.5788	0.5981	0.6206
35	0.3494	0.3739	0.3949	0.4191	0.4595	0.5045	0.5495	0.5899	0.6142	0.6352	0.6596
40	0.3660	0.3926	0.4156	0.4420	0.4861	0.5353	0.5844	0.6285	0.6550	0.6779	0.7046
45	0.3847	0.4139	0.4390	0.4680	0.5163	0.5701	0.6240	0.6723	0.7012	0.7263	0.7555
50	0.4057	0.4377	0.4653	0.4970	0.5500	0.6091	0.6682	0.7212	0.7529	0.7805	0.8125
55	0.4289	0.4640	0.4943	0.5292	0.5873	0.6522	0.7170	0.7752	0.8101	0.8403	0.8755
60	0.4542	0.4928	0.5260	0.5643	0.6282	0.6993	0.7705	0.8344	0.8726	0.9058	0.9444
65	0.4818	0.5241	0.5606	0.6025	0.6726	0.7506	0.8287	0.8987	0.9407	0.9771	1.0194
70	0.5116	0.5580	0.5978	0.6438	0.7205	0.8060	0.8915	0.9681	1.0141	1.0540	1.1003
75	0.5436	0.5943	0.6379	0.6881	0.7720	0.8654	0.9589	1.0428	1.0930	1.1366	1.1873

**Table 7:** Right common carotid intima media thickness [mm] percentiles for healthy male subjects.

Age [years]	1st	2.5th	5th	10th	25th	50th	75th	90th	95th	97.5th	99th
20	0.2929	0.3206	0.3445	0.3720	0.4178	0.4689	0.5201	0.5659	0.5934	0.6172	0.6450
25	0.3000	0.3290	0.3539	0.3826	0.4305	0.4839	0.5373	0.5852	0.6139	0.6388	0.6678
30	0.3087	0.3392	0.3654	0.3956	0.4460	0.5022	0.5584	0.6088	0.6390	0.6652	0.6957
35	0.3190	0.3512	0.3790	0.4110	0.4643	0.5238	0.5833	0.6367	0.6687	0.6964	0.7287
40	0.3308	0.3651	0.3947	0.4287	0.4855	0.5488	0.6120	0.6688	0.7029	0.7324	0.7667
45	0.3442	0.3809	0.4124	0.4488	0.5094	0.5770	0.6446	0.7053	0.7416	0.7732	0.8098
50	0.3592	0.3985	0.4323	0.4712	0.5362	0.6086	0.6810	0.7460	0.7850	0.8187	0.8580
55	0.3758	0.4180	0.4543	0.4961	0.5658	0.6436	0.7213	0.7910	0.8329	0.8691	0.9113
60	0.3940	0.4393	0.4783	0.5232	0.5982	0.6818	0.7654	0.8404	0.8853	0.9243	0.9696
65	0.4137	0.4625	0.5044	0.5528	0.6335	0.7234	0.8133	0.8940	0.9423	0.9843	1.0330
70	0.4350	0.4875	0.5327	0.5847	0.6715	0.7683	0.8650	0.9518	1.0039	1.0490	1.1015

**Table 8:** Right common carotid intima media thickness [mm] percentiles for healthy female subjects.

Age [years]	1st	2.5th	5th	10th	25th	50th	75th	90th	95th	97.5th	99th
25	0.3184	0.3401	0.3588	0.3803	0.4162	0.4562	0.4962	0.5321	0.5536	0.5723	0.5940
30	0.3282	0.3520	0.3725	0.3960	0.4354	0.4792	0.5231	0.5624	0.5860	0.6064	0.6302
35	0.3398	0.3661	0.3886	0.4146	0.4580	0.5064	0.5548	0.5982	0.6242	0.6467	0.6730
40	0.3532	0.3823	0.4073	0.4361	0.4842	0.5378	0.5914	0.6394	0.6683	0.6933	0.7223
45	0.3683	0.4006	0.4284	0.4604	0.5138	0.5733	0.6328	0.6862	0.7182	0.7460	0.7783
50	0.3853	0.4211	0.4520	0.4876	0.5469	0.6130	0.6792	0.7385	0.7741	0.8049	0.8408
55	0.4040	0.4438	0.4781	0.5176	0.5835	0.6569	0.7304	0.7963	0.8358	0.8701	0.9099
60	0.4245	0.4686	0.5066	0.5505	0.6236	0.7050	0.7865	0.8596	0.9034	0.9414	0.9856
65	0.4467	0.4956	0.5377	0.5862	0.6671	0.7573	0.8475	0.9284	0.9769	1.0190	1.0679
70	0.4708	0.5248	0.5713	0.6248	0.7142	0.8137	0.9133	1.0027	1.0562	1.1027	1.1567
75	0.4966	0.5561	0.6073	0.6663	0.7647	0.8744	0.9841	1.0825	1.1415	1.1926	1.2521

**Table 9:** Left common carotid intima media thickness [mm] percentiles for healthy male subjects.

Age [years]	1st	2.5th	5th	10th	25th	50th	75th	90th	95th	97.5th	99th
20	0.3292	0.3533	0.3740	0.3979	0.4378	0.4822	0.5267	0.5665	0.5904	0.6112	0.6353
25	0.3340	0.3601	0.3826	0.4085	0.4517	0.4999	0.5480	0.5912	0.6171	0.6396	0.6657
30	0.3400	0.3685	0.3931	0.4215	0.4688	0.5215	0.5742	0.6215	0.6498	0.6744	0.7030
35	0.3470	0.3785	0.4056	0.4368	0.4889	0.5470	0.6051	0.6572	0.6884	0.7155	0.7470
40	0.3551	0.3899	0.4199	0.4545	0.5122	0.5764	0.6407	0.6984	0.7329	0.7629	0.7978
45	0.3642	0.4029	0.4362	0.4745	0.5385	0.6098	0.6811	0.7451	0.7834	0.8167	0.8553
50	0.3745	0.4174	0.4544	0.4969	0.5679	0.6471	0.7262	0.7972	0.8398	0.8767	0.9197
55	0.3858	0.4335	0.4744	0.5217	0.6005	0.6883	0.7761	0.8549	0.9021	0.9431	0.9908
60	0.3982	0.4510	0.4964	0.5488	0.6361	0.7334	0.8308	0.9181	0.9704	1.0158	1.0686
65	0.4117	0.4701	0.5204	0.5783	0.6748	0.7825	0.8902	0.9867	1.0446	1.0949	1.1533
70	0.4263	0.4908	0.5462	0.6101	0.7167	0.8355	0.9543	1.0609	1.1248	1.1802	1.2447

**Table 10:** Left common carotid intima media thickness [mm] percentiles for healthy female subjects.

Age [years]	1st	2.5th	5th	10th	25th	50th	75th	90th	95th	97.5th	99th
25	0.3063	0.3310	0.3523	0.3768	0.4177	0.4632	0.5088	0.5496	0.5741	0.5954	0.6201
30	0.3186	0.3454	0.3685	0.3951	0.4395	0.4890	0.5385	0.5829	0.6095	0.6326	0.6594
35	0.3331	0.3624	0.3877	0.4168	0.4653	0.5194	0.5736	0.6221	0.6512	0.6765	0.7058
40	0.3498	0.3820	0.4098	0.4418	0.4951	0.5546	0.6141	0.6674	0.6994	0.7271	0.7594
45	0.3687	0.4043	0.4348	0.4701	0.5289	0.5944	0.6599	0.7187	0.7540	0.7846	0.8201
50	0.3899	0.4291	0.4628	0.5017	0.5666	0.6389	0.7112	0.7761	0.8150	0.8488	0.8880
55	0.4133	0.4565	0.4938	0.5367	0.6083	0.6881	0.7679	0.8395	0.8825	0.9197	0.9630
60	0.4389	0.4866	0.5277	0.5750	0.6540	0.7420	0.8300	0.9090	0.9563	0.9974	1.0451
65	0.4667	0.5193	0.5645	0.6167	0.7036	0.8006	0.8975	0.9845	1.0366	1.0818	1.1344
70	0.4968	0.5546	0.6043	0.6616	0.7572	0.8638	0.9704	1.0660	1.1233	1.1730	1.2308
75	0.5291	0.5925	0.6471	0.7099	0.8148	0.9318	1.0487	1.1536	1.2165	1.2710	1.3344

## Supplementary Materials

Supplementary Materials Table A: averaged (left and right) CIMT [mm] percentiles for healthy male subjects. Table B: averaged (left and right) CIMT [mm] percentiles for healthy female subjects. Table C: right CIMT [mm] percentiles for healthy male subjects. Table D: right CIMT [mm] percentiles for healthy female subjects. Table E: left CIMT [mm] percentiles for healthy male subjects. Table F: left CIMT [mm] percentiles for healthy female subjects.
