# Supplementary material for: Carotid Intima Media Thickness Reference Intervals for a Healthy Argentinean Population Aged 11–81 Years
Source: Int J Hypertens. 2018 Feb 14;2018:8086714. doi: 10.1155/2018/8086714 (PMC5832113; doi:10.1155/2018/8086714)
Supplement: Supplementary Materials — Table A: averaged (left and right) CIMT [mm] percentiles for healthy male subjects. Table B: averaged (left and right) CIMT [mm] percentiles for healthy female subjects. Table C: right CIMT [mm] percentiles for healthy male subjects. Table D: right CIMT [mm] percentiles for healthy female subjects. Table E: left CIMT [mm] percentiles for healthy male subjects. Table F: left CIMT [mm] percentiles for healthy female subjects. [file 8086714.f1.doc]

- Supplementary material (online only) −

**Carotid intima-media thickness reference intervals for a healthy Argentinean population aged 11-81 years**

Authors: AlejandroDiaz, Daniel Bia, Yanina Zócalo, Hugo Manterola, Ignacio Larrabide, Lucas Lo Vercio, Mariana Del Fresno, Edmundo Cabrera Fischer.

**Results:**

| **Table A. Averaged (Left and Right) CIMT [mm] percentiles for MALE healthy subjects** | | | | | | | | | | | | | |
| --- | --- | --- | --- | --- | --- | --- | --- | --- | --- | --- | --- | --- | --- |
|  |  |  |  |  |  |  |  |  |  |  |  |  |  |
| **Age [years]** | **1 th** | **2.5 th** | **5 th** | **10 th** | **25 th** | **50 th** | **75 th** | **90 th** | **95 th** | **97.5 th** | **99 th** |  |  |
| 11 | 0.2854 | 0.3075 | 0.3266 | 0.3485 | 0.3850 | 0.4258 | 0.4666 | 0.5031 | 0.5250 | 0.5440 | 0.5662 |  |  |
| 12 | 0.2867 | 0.3089 | 0.3280 | 0.3500 | 0.3867 | 0.4277 | 0.4686 | 0.5053 | 0.5273 | 0.5464 | 0.5686 |  |  |
| 13 | 0.2881 | 0.3104 | 0.3296 | 0.3517 | 0.3886 | 0.4297 | 0.4708 | 0.5077 | 0.5298 | 0.5490 | 0.5713 |  |  |
| 14 | 0.2896 | 0.3120 | 0.3313 | 0.3535 | 0.3906 | 0.4319 | 0.4732 | 0.5103 | 0.5325 | 0.5518 | 0.5742 |  |  |
| 15 | 0.2913 | 0.3138 | 0.3332 | 0.3555 | 0.3927 | 0.4343 | 0.4758 | 0.5130 | 0.5354 | 0.5547 | 0.5773 |  |  |
| 16 | 0.2930 | 0.3156 | 0.3351 | 0.3576 | 0.3950 | 0.4368 | 0.4785 | 0.5160 | 0.5385 | 0.5579 | 0.5806 |  |  |
| 17 | 0.2948 | 0.3176 | 0.3372 | 0.3598 | 0.3975 | 0.4395 | 0.4815 | 0.5191 | 0.5417 | 0.5613 | 0.5841 |  |  |
| 18 | 0.2968 | 0.3197 | 0.3394 | 0.3622 | 0.4001 | 0.4423 | 0.4846 | 0.5225 | 0.5452 | 0.5649 | 0.5878 |  |  |
| 19 | 0.2989 | 0.3219 | 0.3418 | 0.3646 | 0.4028 | 0.4453 | 0.4879 | 0.5260 | 0.5489 | 0.5687 | 0.5918 |  |  |
| 20 | 0.3011 | 0.3243 | 0.3442 | 0.3673 | 0.4057 | 0.4485 | 0.4913 | 0.5297 | 0.5528 | 0.5727 | 0.5960 |  |  |
| 21 | 0.3033 | 0.3267 | 0.3468 | 0.3700 | 0.4087 | 0.4518 | 0.4950 | 0.5337 | 0.5568 | 0.5770 | 0.6003 |  |  |
| 22 | 0.3058 | 0.3293 | 0.3496 | 0.3729 | 0.4119 | 0.4553 | 0.4988 | 0.5378 | 0.5611 | 0.5814 | 0.6049 |  |  |
| 23 | 0.3083 | 0.3320 | 0.3524 | 0.3760 | 0.4152 | 0.4590 | 0.5028 | 0.5420 | 0.5656 | 0.5860 | 0.6097 |  |  |
| 24 | 0.3109 | 0.3348 | 0.3554 | 0.3791 | 0.4187 | 0.4628 | 0.5070 | 0.5465 | 0.5703 | 0.5908 | 0.6148 |  |  |
| 25 | 0.3136 | 0.3378 | 0.3585 | 0.3824 | 0.4223 | 0.4668 | 0.5113 | 0.5512 | 0.5751 | 0.5959 | 0.6200 |  |  |
| 26 | 0.3165 | 0.3408 | 0.3618 | 0.3859 | 0.4261 | 0.4710 | 0.5158 | 0.5561 | 0.5802 | 0.6011 | 0.6255 |  |  |
| 27 | 0.3195 | 0.3440 | 0.3651 | 0.3894 | 0.4300 | 0.4753 | 0.5205 | 0.5611 | 0.5855 | 0.6066 | 0.6311 |  |  |
| 28 | 0.3225 | 0.3473 | 0.3686 | 0.3932 | 0.4341 | 0.4798 | 0.5254 | 0.5664 | 0.5909 | 0.6122 | 0.6370 |  |  |
| 29 | 0.3257 | 0.3507 | 0.3722 | 0.3970 | 0.4383 | 0.4844 | 0.5305 | 0.5718 | 0.5966 | 0.6181 | 0.6431 |  |  |
| 30 | 0.3290 | 0.3543 | 0.3760 | 0.4010 | 0.4427 | 0.4892 | 0.5357 | 0.5774 | 0.6025 | 0.6242 | 0.6494 |  |  |
| 31 | 0.3324 | 0.3579 | 0.3798 | 0.4051 | 0.4472 | 0.4942 | 0.5411 | 0.5833 | 0.6085 | 0.6304 | 0.6559 |  |  |
| 32 | 0.3360 | 0.3617 | 0.3838 | 0.4093 | 0.4519 | 0.4993 | 0.5467 | 0.5893 | 0.6148 | 0.6369 | 0.6626 |  |  |
| 33 | 0.3396 | 0.3656 | 0.3879 | 0.4137 | 0.4567 | 0.5046 | 0.5525 | 0.5955 | 0.6212 | 0.6436 | 0.6696 |  |  |
| 34 | 0.3434 | 0.3696 | 0.3922 | 0.4182 | 0.4616 | 0.5100 | 0.5584 | 0.6019 | 0.6279 | 0.6505 | 0.6767 |  |  |
| 35 | 0.3472 | 0.3738 | 0.3966 | 0.4229 | 0.4668 | 0.5157 | 0.5646 | 0.6085 | 0.6348 | 0.6576 | 0.6841 |  |  |
| 36 | 0.3512 | 0.3780 | 0.4011 | 0.4277 | 0.4720 | 0.5214 | 0.5709 | 0.6152 | 0.6418 | 0.6649 | 0.6917 |  |  |
| 37 | 0.3553 | 0.3824 | 0.4057 | 0.4326 | 0.4774 | 0.5274 | 0.5774 | 0.6222 | 0.6491 | 0.6724 | 0.6995 |  |  |
| 38 | 0.3595 | 0.3869 | 0.4105 | 0.4376 | 0.4830 | 0.5335 | 0.5840 | 0.6294 | 0.6565 | 0.6801 | 0.7075 |  |  |
| 39 | 0.3638 | 0.3915 | 0.4153 | 0.4428 | 0.4887 | 0.5398 | 0.5909 | 0.6367 | 0.6642 | 0.6880 | 0.7157 |  |  |
| 40 | 0.3682 | 0.3962 | 0.4203 | 0.4481 | 0.4945 | 0.5462 | 0.5979 | 0.6442 | 0.6720 | 0.6961 | 0.7242 |  |  |
| 41 | 0.3727 | 0.4011 | 0.4255 | 0.4536 | 0.5005 | 0.5528 | 0.6051 | 0.6520 | 0.6801 | 0.7045 | 0.7328 |  |  |
| 42 | 0.3774 | 0.4061 | 0.4307 | 0.4592 | 0.5066 | 0.5595 | 0.6124 | 0.6599 | 0.6883 | 0.7130 | 0.7417 |  |  |
| 43 | 0.3821 | 0.4112 | 0.4361 | 0.4649 | 0.5129 | 0.5665 | 0.6200 | 0.6680 | 0.6968 | 0.7218 | 0.7508 |  |  |
| 44 | 0.3870 | 0.4164 | 0.4417 | 0.4708 | 0.5194 | 0.5735 | 0.6277 | 0.6763 | 0.7054 | 0.7307 | 0.7601 |  |  |
| 45 | 0.3920 | 0.4217 | 0.4473 | 0.4768 | 0.5260 | 0.5808 | 0.6356 | 0.6848 | 0.7143 | 0.7398 | 0.7696 |  |  |
| 46 | 0.3971 | 0.4272 | 0.4531 | 0.4829 | 0.5327 | 0.5882 | 0.6437 | 0.6935 | 0.7233 | 0.7492 | 0.7793 |  |  |
| 47 | 0.4023 | 0.4328 | 0.4590 | 0.4892 | 0.5396 | 0.5958 | 0.6519 | 0.7023 | 0.7326 | 0.7588 | 0.7892 |  |  |
| 48 | 0.4076 | 0.4385 | 0.4650 | 0.4956 | 0.5466 | 0.6035 | 0.6604 | 0.7114 | 0.7420 | 0.7685 | 0.7994 |  |  |
| 49 | 0.4130 | 0.4443 | 0.4711 | 0.5021 | 0.5538 | 0.6114 | 0.6690 | 0.7207 | 0.7516 | 0.7785 | 0.8098 |  |  |
| 50 | 0.4186 | 0.4502 | 0.4774 | 0.5088 | 0.5611 | 0.6195 | 0.6778 | 0.7301 | 0.7615 | 0.7887 | 0.8203 |  |  |
| 51 | 0.4242 | 0.4563 | 0.4838 | 0.5156 | 0.5686 | 0.6277 | 0.6868 | 0.7398 | 0.7715 | 0.7991 | 0.8311 |  |  |
| 52 | 0.4300 | 0.4624 | 0.4904 | 0.5225 | 0.5762 | 0.6361 | 0.6959 | 0.7496 | 0.7818 | 0.8097 | 0.8421 |  |  |
| 53 | 0.4359 | 0.4687 | 0.4970 | 0.5296 | 0.5840 | 0.6446 | 0.7052 | 0.7596 | 0.7922 | 0.8205 | 0.8533 |  |  |
| 54 | 0.4419 | 0.4752 | 0.5038 | 0.5368 | 0.5919 | 0.6533 | 0.7147 | 0.7698 | 0.8028 | 0.8315 | 0.8648 |  |  |
| 55 | 0.4480 | 0.4817 | 0.5107 | 0.5442 | 0.6000 | 0.6622 | 0.7244 | 0.7802 | 0.8137 | 0.8427 | 0.8764 |  |  |
| 56 | 0.4542 | 0.4884 | 0.5178 | 0.5517 | 0.6082 | 0.6712 | 0.7343 | 0.7908 | 0.8247 | 0.8541 | 0.8883 |  |  |
| 57 | 0.4605 | 0.4951 | 0.5249 | 0.5593 | 0.6166 | 0.6804 | 0.7443 | 0.8016 | 0.8359 | 0.8657 | 0.9004 |  |  |
| 58 | 0.4669 | 0.5020 | 0.5322 | 0.5670 | 0.6251 | 0.6898 | 0.7545 | 0.8126 | 0.8474 | 0.8775 | 0.9126 |  |  |
| 59 | 0.4735 | 0.5090 | 0.5396 | 0.5749 | 0.6337 | 0.6993 | 0.7649 | 0.8237 | 0.8590 | 0.8896 | 0.9251 |  |  |
| 60 | 0.4801 | 0.5162 | 0.5472 | 0.5829 | 0.6425 | 0.7090 | 0.7755 | 0.8351 | 0.8708 | 0.9018 | 0.9379 |  |  |
| 61 | 0.4869 | 0.5234 | 0.5549 | 0.5911 | 0.6515 | 0.7188 | 0.7862 | 0.8466 | 0.8828 | 0.9143 | 0.9508 |  |  |
| 62 | 0.4938 | 0.5308 | 0.5627 | 0.5994 | 0.6606 | 0.7289 | 0.7971 | 0.8584 | 0.8951 | 0.9269 | 0.9639 |  |  |
| 63 | 0.5008 | 0.5383 | 0.5706 | 0.6078 | 0.6699 | 0.7390 | 0.8082 | 0.8703 | 0.9075 | 0.9398 | 0.9773 |  |  |
| 64 | 0.5079 | 0.5459 | 0.5786 | 0.6163 | 0.6793 | 0.7494 | 0.8195 | 0.8824 | 0.9201 | 0.9528 | 0.9909 |  |  |
| 65 | 0.5151 | 0.5537 | 0.5868 | 0.6250 | 0.6888 | 0.7599 | 0.8309 | 0.8947 | 0.9329 | 0.9661 | 1.0046 |  |  |
| 66 | 0.5224 | 0.5615 | 0.5951 | 0.6339 | 0.6985 | 0.7705 | 0.8426 | 0.9072 | 0.9460 | 0.9796 | 1.0186 |  |  |
| 67 | 0.5299 | 0.5695 | 0.6036 | 0.6428 | 0.7083 | 0.7814 | 0.8544 | 0.9199 | 0.9592 | 0.9932 | 1.0328 |  |  |
| 68 | 0.5374 | 0.5776 | 0.6121 | 0.6519 | 0.7183 | 0.7924 | 0.8664 | 0.9328 | 0.9726 | 1.0071 | 1.0473 |  |  |
| 69 | 0.5451 | 0.5858 | 0.6208 | 0.6612 | 0.7285 | 0.8035 | 0.8785 | 0.9459 | 0.9862 | 1.0212 | 1.0619 |  |  |
| 70 | 0.5529 | 0.5941 | 0.6296 | 0.6705 | 0.7388 | 0.8148 | 0.8909 | 0.9591 | 1.0000 | 1.0355 | 1.0768 |  |  |
| 71 | 0.5608 | 0.6026 | 0.6386 | 0.6800 | 0.7492 | 0.8263 | 0.9034 | 0.9726 | 1.0140 | 1.0500 | 1.0918 |  |  |
| 72 | 0.5688 | 0.6112 | 0.6476 | 0.6897 | 0.7598 | 0.8379 | 0.9161 | 0.9862 | 1.0283 | 1.0647 | 1.1071 |  |  |
| 73 | 0.5769 | 0.6199 | 0.6568 | 0.6994 | 0.7705 | 0.8497 | 0.9290 | 1.0001 | 1.0427 | 1.0796 | 1.1226 |  |  |
| 74 | 0.5851 | 0.6287 | 0.6661 | 0.7093 | 0.7814 | 0.8617 | 0.9420 | 1.0141 | 1.0573 | 1.0947 | 1.1383 |  |  |
| 75 | 0.5935 | 0.6376 | 0.6756 | 0.7194 | 0.7924 | 0.8738 | 0.9553 | 1.0283 | 1.0721 | 1.1101 | 1.1542 |  |  |
| 76 | 0.6019 | 0.6467 | 0.6852 | 0.7296 | 0.8036 | 0.8861 | 0.9687 | 1.0427 | 1.0871 | 1.1256 | 1.1704 |  |  |
| 77 | 0.6105 | 0.6558 | 0.6949 | 0.7399 | 0.8149 | 0.8986 | 0.9822 | 1.0573 | 1.1023 | 1.1413 | 1.1867 |  |  |
| 78 | 0.6191 | 0.6651 | 0.7047 | 0.7503 | 0.8264 | 0.9112 | 0.9960 | 1.0721 | 1.1177 | 1.1573 | 1.2033 |  |  |
| 79 | 0.6279 | 0.6746 | 0.7147 | 0.7609 | 0.8380 | 0.9240 | 1.0099 | 1.0871 | 1.1333 | 1.1734 | 1.2200 |  |  |
| 80 | 0.6368 | 0.6841 | 0.7247 | 0.7716 | 0.8498 | 0.9369 | 1.0241 | 1.1022 | 1.1491 | 1.1898 | 1.2370 |  |  |
| 81 | 0.6458 | 0.6937 | 0.7349 | 0.7825 | 0.8617 | 0.9500 | 1.0384 | 1.1176 | 1.1651 | 1.2063 | 1.2542 |  |  |

| **Table B. Averaged (Left and Right) CIMT [mm] percentiles for FEMALE healthy subjects** | | | | | | | | | | | | | |
| --- | --- | --- | --- | --- | --- | --- | --- | --- | --- | --- | --- | --- | --- |
|  |  |  |  |  |  |  |  |  |  |  |  |  |  |
| **Age [years]** | **1 th** | **2.5 th** | **5 th** | **10 th** | **25 th** | **50 th** | **75 th** | **90 th** | **95 th** | **97.5 th** | **99 th** |  |  |
| 11 | 0.3007 | 0.3186 | 0.3339 | 0.3516 | 0.3811 | 0.4139 | 0.4468 | 0.4763 | 0.4940 | 0.5093 | 0.5272 |  |  |
| 12 | 0.3017 | 0.3197 | 0.3352 | 0.3530 | 0.3827 | 0.4158 | 0.4490 | 0.4787 | 0.4965 | 0.5120 | 0.5299 |  |  |
| 13 | 0.3028 | 0.3210 | 0.3365 | 0.3545 | 0.3845 | 0.4179 | 0.4513 | 0.4813 | 0.4992 | 0.5148 | 0.5329 |  |  |
| 14 | 0.3040 | 0.3223 | 0.3380 | 0.3562 | 0.3864 | 0.4201 | 0.4538 | 0.4840 | 0.5022 | 0.5179 | 0.5362 |  |  |
| 15 | 0.3053 | 0.3238 | 0.3396 | 0.3579 | 0.3885 | 0.4225 | 0.4565 | 0.4870 | 0.5053 | 0.5212 | 0.5397 |  |  |
| 16 | 0.3067 | 0.3253 | 0.3413 | 0.3598 | 0.3907 | 0.4250 | 0.4594 | 0.4902 | 0.5087 | 0.5247 | 0.5434 |  |  |
| 17 | 0.3081 | 0.3270 | 0.3432 | 0.3618 | 0.3930 | 0.4277 | 0.4625 | 0.4936 | 0.5123 | 0.5285 | 0.5473 |  |  |
| 18 | 0.3097 | 0.3287 | 0.3451 | 0.3640 | 0.3955 | 0.4306 | 0.4657 | 0.4972 | 0.5161 | 0.5325 | 0.5515 |  |  |
| 19 | 0.3113 | 0.3306 | 0.3471 | 0.3662 | 0.3981 | 0.4336 | 0.4692 | 0.5010 | 0.5201 | 0.5367 | 0.5560 |  |  |
| 20 | 0.3130 | 0.3325 | 0.3493 | 0.3686 | 0.4009 | 0.4368 | 0.4728 | 0.5050 | 0.5244 | 0.5411 | 0.5606 |  |  |
| 21 | 0.3148 | 0.3346 | 0.3516 | 0.3711 | 0.4038 | 0.4402 | 0.4766 | 0.5093 | 0.5288 | 0.5458 | 0.5656 |  |  |
| 22 | 0.3167 | 0.3367 | 0.3539 | 0.3738 | 0.4069 | 0.4437 | 0.4806 | 0.5137 | 0.5335 | 0.5507 | 0.5707 |  |  |
| 23 | 0.3187 | 0.3390 | 0.3564 | 0.3765 | 0.4100 | 0.4474 | 0.4848 | 0.5183 | 0.5384 | 0.5558 | 0.5761 |  |  |
| 24 | 0.3208 | 0.3413 | 0.3590 | 0.3794 | 0.4134 | 0.4513 | 0.4892 | 0.5232 | 0.5435 | 0.5612 | 0.5818 |  |  |
| 25 | 0.3230 | 0.3438 | 0.3617 | 0.3824 | 0.4169 | 0.4553 | 0.4937 | 0.5282 | 0.5489 | 0.5668 | 0.5876 |  |  |
| 26 | 0.3252 | 0.3464 | 0.3645 | 0.3855 | 0.4205 | 0.4595 | 0.4985 | 0.5334 | 0.5544 | 0.5726 | 0.5937 |  |  |
| 27 | 0.3275 | 0.3490 | 0.3675 | 0.3888 | 0.4243 | 0.4638 | 0.5034 | 0.5389 | 0.5602 | 0.5786 | 0.6001 |  |  |
| 28 | 0.3300 | 0.3518 | 0.3705 | 0.3921 | 0.4282 | 0.4683 | 0.5085 | 0.5446 | 0.5662 | 0.5849 | 0.6067 |  |  |
| 29 | 0.3325 | 0.3546 | 0.3737 | 0.3956 | 0.4322 | 0.4730 | 0.5138 | 0.5504 | 0.5724 | 0.5914 | 0.6135 |  |  |
| 30 | 0.3351 | 0.3576 | 0.3769 | 0.3992 | 0.4364 | 0.4779 | 0.5193 | 0.5565 | 0.5788 | 0.5981 | 0.6206 |  |  |
| 31 | 0.3378 | 0.3606 | 0.3803 | 0.4029 | 0.4407 | 0.4829 | 0.5250 | 0.5628 | 0.5854 | 0.6051 | 0.6279 |  |  |
| 32 | 0.3406 | 0.3638 | 0.3838 | 0.4068 | 0.4452 | 0.4880 | 0.5308 | 0.5693 | 0.5923 | 0.6123 | 0.6355 |  |  |
| 33 | 0.3434 | 0.3670 | 0.3874 | 0.4108 | 0.4498 | 0.4934 | 0.5369 | 0.5759 | 0.5994 | 0.6197 | 0.6433 |  |  |
| 34 | 0.3464 | 0.3704 | 0.3911 | 0.4149 | 0.4546 | 0.4989 | 0.5431 | 0.5828 | 0.6067 | 0.6273 | 0.6513 |  |  |
| 35 | 0.3494 | 0.3739 | 0.3949 | 0.4191 | 0.4595 | 0.5045 | 0.5495 | 0.5899 | 0.6142 | 0.6352 | 0.6596 |  |  |
| 36 | 0.3526 | 0.3774 | 0.3988 | 0.4234 | 0.4645 | 0.5103 | 0.5561 | 0.5972 | 0.6219 | 0.6433 | 0.6681 |  |  |
| 37 | 0.3558 | 0.3811 | 0.4028 | 0.4279 | 0.4697 | 0.5163 | 0.5629 | 0.6048 | 0.6298 | 0.6516 | 0.6769 |  |  |
| 38 | 0.3591 | 0.3848 | 0.4070 | 0.4325 | 0.4750 | 0.5225 | 0.5699 | 0.6125 | 0.6380 | 0.6601 | 0.6859 |  |  |
| 39 | 0.3625 | 0.3887 | 0.4112 | 0.4372 | 0.4805 | 0.5288 | 0.5771 | 0.6204 | 0.6464 | 0.6689 | 0.6951 |  |  |
| 40 | 0.3660 | 0.3926 | 0.4156 | 0.4420 | 0.4861 | 0.5353 | 0.5844 | 0.6285 | 0.6550 | 0.6779 | 0.7046 |  |  |
| 41 | 0.3696 | 0.3967 | 0.4200 | 0.4470 | 0.4919 | 0.5419 | 0.5920 | 0.6369 | 0.6638 | 0.6871 | 0.7143 |  |  |
| 42 | 0.3732 | 0.4009 | 0.4246 | 0.4520 | 0.4978 | 0.5487 | 0.5997 | 0.6454 | 0.6728 | 0.6966 | 0.7242 |  |  |
| 43 | 0.3770 | 0.4051 | 0.4293 | 0.4572 | 0.5038 | 0.5557 | 0.6076 | 0.6542 | 0.6821 | 0.7063 | 0.7344 |  |  |
| 44 | 0.3808 | 0.4095 | 0.4341 | 0.4626 | 0.5100 | 0.5628 | 0.6157 | 0.6631 | 0.6915 | 0.7162 | 0.7449 |  |  |
| 45 | 0.3847 | 0.4139 | 0.4390 | 0.4680 | 0.5163 | 0.5701 | 0.6240 | 0.6723 | 0.7012 | 0.7263 | 0.7555 |  |  |
| 46 | 0.3888 | 0.4185 | 0.4441 | 0.4736 | 0.5228 | 0.5776 | 0.6324 | 0.6816 | 0.7111 | 0.7367 | 0.7665 |  |  |
| 47 | 0.3929 | 0.4232 | 0.4492 | 0.4793 | 0.5294 | 0.5852 | 0.6411 | 0.6912 | 0.7212 | 0.7473 | 0.7776 |  |  |
| 48 | 0.3970 | 0.4279 | 0.4545 | 0.4851 | 0.5361 | 0.5930 | 0.6499 | 0.7010 | 0.7316 | 0.7581 | 0.7890 |  |  |
| 49 | 0.4013 | 0.4328 | 0.4598 | 0.4910 | 0.5430 | 0.6010 | 0.6590 | 0.7110 | 0.7421 | 0.7692 | 0.8006 |  |  |
| 50 | 0.4057 | 0.4377 | 0.4653 | 0.4970 | 0.5500 | 0.6091 | 0.6682 | 0.7212 | 0.7529 | 0.7805 | 0.8125 |  |  |
| 51 | 0.4102 | 0.4428 | 0.4709 | 0.5032 | 0.5572 | 0.6174 | 0.6776 | 0.7315 | 0.7639 | 0.7920 | 0.8246 |  |  |
| 52 | 0.4147 | 0.4480 | 0.4766 | 0.5095 | 0.5645 | 0.6258 | 0.6871 | 0.7421 | 0.7751 | 0.8037 | 0.8370 |  |  |
| 53 | 0.4193 | 0.4532 | 0.4824 | 0.5159 | 0.5720 | 0.6344 | 0.6969 | 0.7530 | 0.7865 | 0.8157 | 0.8496 |  |  |
| 54 | 0.4241 | 0.4586 | 0.4883 | 0.5225 | 0.5796 | 0.6432 | 0.7069 | 0.7640 | 0.7982 | 0.8279 | 0.8624 |  |  |
| 55 | 0.4289 | 0.4640 | 0.4943 | 0.5292 | 0.5873 | 0.6522 | 0.7170 | 0.7752 | 0.8101 | 0.8403 | 0.8755 |  |  |
| 56 | 0.4338 | 0.4696 | 0.5004 | 0.5359 | 0.5952 | 0.6613 | 0.7273 | 0.7866 | 0.8221 | 0.8530 | 0.8888 |  |  |
| 57 | 0.4387 | 0.4753 | 0.5067 | 0.5429 | 0.6032 | 0.6705 | 0.7378 | 0.7982 | 0.8344 | 0.8658 | 0.9023 |  |  |
| 58 | 0.4438 | 0.4810 | 0.5130 | 0.5499 | 0.6114 | 0.6800 | 0.7485 | 0.8101 | 0.8469 | 0.8789 | 0.9161 |  |  |
| 59 | 0.4490 | 0.4869 | 0.5195 | 0.5570 | 0.6197 | 0.6896 | 0.7594 | 0.8221 | 0.8597 | 0.8923 | 0.9302 |  |  |
| 60 | 0.4542 | 0.4928 | 0.5260 | 0.5643 | 0.6282 | 0.6993 | 0.7705 | 0.8344 | 0.8726 | 0.9058 | 0.9444 |  |  |
| 61 | 0.4596 | 0.4989 | 0.5327 | 0.5717 | 0.6368 | 0.7093 | 0.7818 | 0.8468 | 0.8858 | 0.9196 | 0.9589 |  |  |
| 62 | 0.4650 | 0.5051 | 0.5395 | 0.5792 | 0.6455 | 0.7194 | 0.7932 | 0.8595 | 0.8992 | 0.9336 | 0.9737 |  |  |
| 63 | 0.4705 | 0.5113 | 0.5464 | 0.5869 | 0.6544 | 0.7296 | 0.8048 | 0.8723 | 0.9128 | 0.9479 | 0.9887 |  |  |
| 64 | 0.4761 | 0.5177 | 0.5534 | 0.5946 | 0.6634 | 0.7400 | 0.8167 | 0.8854 | 0.9266 | 0.9624 | 1.0039 |  |  |
| 65 | 0.4818 | 0.5241 | 0.5606 | 0.6025 | 0.6726 | 0.7506 | 0.8287 | 0.8987 | 0.9407 | 0.9771 | 1.0194 |  |  |
| 66 | 0.4876 | 0.5307 | 0.5678 | 0.6105 | 0.6819 | 0.7614 | 0.8408 | 0.9122 | 0.9549 | 0.9920 | 1.0351 |  |  |
| 67 | 0.4935 | 0.5374 | 0.5751 | 0.6187 | 0.6913 | 0.7723 | 0.8532 | 0.9258 | 0.9694 | 1.0071 | 1.0511 |  |  |
| 68 | 0.4994 | 0.5441 | 0.5826 | 0.6269 | 0.7009 | 0.7833 | 0.8658 | 0.9397 | 0.9841 | 1.0225 | 1.0672 |  |  |
| 69 | 0.5055 | 0.5510 | 0.5902 | 0.6353 | 0.7106 | 0.7946 | 0.8785 | 0.9538 | 0.9990 | 1.0381 | 1.0837 |  |  |
| 70 | 0.5116 | 0.5580 | 0.5978 | 0.6438 | 0.7205 | 0.8060 | 0.8915 | 0.9681 | 1.0141 | 1.0540 | 1.1003 |  |  |
| 71 | 0.5178 | 0.5650 | 0.6056 | 0.6524 | 0.7305 | 0.8175 | 0.9046 | 0.9827 | 1.0295 | 1.0701 | 1.1173 |  |  |
| 72 | 0.5241 | 0.5722 | 0.6135 | 0.6612 | 0.7407 | 0.8293 | 0.9179 | 0.9974 | 1.0450 | 1.0864 | 1.1344 |  |  |
| 73 | 0.5305 | 0.5795 | 0.6215 | 0.6700 | 0.7510 | 0.8412 | 0.9314 | 1.0123 | 1.0608 | 1.1029 | 1.1518 |  |  |
| 74 | 0.5370 | 0.5868 | 0.6297 | 0.6790 | 0.7614 | 0.8532 | 0.9450 | 1.0274 | 1.0768 | 1.1196 | 1.1694 |  |  |
| 75 | 0.5436 | 0.5943 | 0.6379 | 0.6881 | 0.7720 | 0.8654 | 0.9589 | 1.0428 | 1.0930 | 1.1366 | 1.1873 |  |  |
| 76 | 0.5503 | 0.6019 | 0.6462 | 0.6974 | 0.7827 | 0.8778 | 0.9730 | 1.0583 | 1.1094 | 1.1538 | 1.2054 |  |  |
| 77 | 0.5570 | 0.6095 | 0.6547 | 0.7067 | 0.7936 | 0.8904 | 0.9872 | 1.0740 | 1.1261 | 1.1713 | 1.2238 |  |  |
| 78 | 0.5639 | 0.6173 | 0.6632 | 0.7162 | 0.8046 | 0.9031 | 1.0016 | 1.0900 | 1.1430 | 1.1889 | 1.2423 |  |  |

| **Table C. Right CIMT [mm] percentiles for MALE healthy subjects** | | | | | | | | | | | |
| --- | --- | --- | --- | --- | --- | --- | --- | --- | --- | --- | --- |
|  |  |  |  |  |  |  |  |  |  |  |  |
| **Age [years]** | **1 th** | **2.5 th** | **5 th** | **10 th** | **25 th** | **50 th** | **75 th** | **90 th** | **95 th** | **97.5 th** | **99 th** |
| 20 | 0.2929 | 0.3206 | 0.3445 | 0.3720 | 0.4178 | 0.4689 | 0.5201 | 0.5659 | 0.5934 | 0.6172 | 0.6450 |
| 21 | 0.2942 | 0.3222 | 0.3462 | 0.3739 | 0.4201 | 0.4717 | 0.5232 | 0.5694 | 0.5971 | 0.6212 | 0.6491 |
| 22 | 0.2956 | 0.3238 | 0.3480 | 0.3759 | 0.4226 | 0.4745 | 0.5265 | 0.5731 | 0.6011 | 0.6253 | 0.6535 |
| 23 | 0.2970 | 0.3254 | 0.3499 | 0.3781 | 0.4251 | 0.4775 | 0.5299 | 0.5770 | 0.6052 | 0.6296 | 0.6581 |
| 24 | 0.2985 | 0.3272 | 0.3518 | 0.3803 | 0.4278 | 0.4807 | 0.5335 | 0.5810 | 0.6095 | 0.6341 | 0.6628 |
| 25 | 0.3000 | 0.3290 | 0.3539 | 0.3826 | 0.4305 | 0.4839 | 0.5373 | 0.5852 | 0.6139 | 0.6388 | 0.6678 |
| 26 | 0.3016 | 0.3309 | 0.3560 | 0.3850 | 0.4334 | 0.4873 | 0.5412 | 0.5896 | 0.6186 | 0.6437 | 0.6730 |
| 27 | 0.3033 | 0.3328 | 0.3582 | 0.3875 | 0.4364 | 0.4908 | 0.5453 | 0.5941 | 0.6234 | 0.6488 | 0.6783 |
| 28 | 0.3050 | 0.3349 | 0.3605 | 0.3901 | 0.4395 | 0.4945 | 0.5495 | 0.5988 | 0.6284 | 0.6541 | 0.6839 |
| 29 | 0.3068 | 0.3370 | 0.3629 | 0.3928 | 0.4427 | 0.4983 | 0.5539 | 0.6037 | 0.6336 | 0.6596 | 0.6897 |
| 30 | 0.3087 | 0.3392 | 0.3654 | 0.3956 | 0.4460 | 0.5022 | 0.5584 | 0.6088 | 0.6390 | 0.6652 | 0.6957 |
| 31 | 0.3106 | 0.3414 | 0.3679 | 0.3985 | 0.4495 | 0.5063 | 0.5631 | 0.6140 | 0.6446 | 0.6711 | 0.7019 |
| 32 | 0.3126 | 0.3438 | 0.3706 | 0.4015 | 0.4530 | 0.5105 | 0.5679 | 0.6194 | 0.6503 | 0.6771 | 0.7083 |
| 33 | 0.3147 | 0.3462 | 0.3733 | 0.4045 | 0.4567 | 0.5148 | 0.5729 | 0.6250 | 0.6563 | 0.6834 | 0.7149 |
| 34 | 0.3168 | 0.3487 | 0.3761 | 0.4077 | 0.4605 | 0.5192 | 0.5780 | 0.6308 | 0.6624 | 0.6898 | 0.7217 |
| 35 | 0.3190 | 0.3512 | 0.3790 | 0.4110 | 0.4643 | 0.5238 | 0.5833 | 0.6367 | 0.6687 | 0.6964 | 0.7287 |
| 36 | 0.3212 | 0.3539 | 0.3820 | 0.4143 | 0.4683 | 0.5285 | 0.5887 | 0.6428 | 0.6751 | 0.7032 | 0.7359 |
| 37 | 0.3235 | 0.3566 | 0.3850 | 0.4178 | 0.4725 | 0.5334 | 0.5943 | 0.6490 | 0.6818 | 0.7102 | 0.7433 |
| 38 | 0.3259 | 0.3594 | 0.3881 | 0.4213 | 0.4767 | 0.5384 | 0.6001 | 0.6554 | 0.6886 | 0.7174 | 0.7509 |
| 39 | 0.3283 | 0.3622 | 0.3914 | 0.4250 | 0.4810 | 0.5435 | 0.6060 | 0.6621 | 0.6957 | 0.7248 | 0.7587 |
| 40 | 0.3308 | 0.3651 | 0.3947 | 0.4287 | 0.4855 | 0.5488 | 0.6120 | 0.6688 | 0.7029 | 0.7324 | 0.7667 |
| 41 | 0.3334 | 0.3681 | 0.3981 | 0.4325 | 0.4900 | 0.5542 | 0.6183 | 0.6758 | 0.7103 | 0.7402 | 0.7749 |
| 42 | 0.3360 | 0.3712 | 0.4015 | 0.4365 | 0.4947 | 0.5597 | 0.6246 | 0.6829 | 0.7178 | 0.7481 | 0.7833 |
| 43 | 0.3387 | 0.3744 | 0.4051 | 0.4405 | 0.4995 | 0.5653 | 0.6311 | 0.6902 | 0.7256 | 0.7563 | 0.7920 |
| 44 | 0.3414 | 0.3776 | 0.4087 | 0.4446 | 0.5044 | 0.5711 | 0.6378 | 0.6976 | 0.7335 | 0.7646 | 0.8008 |
| 45 | 0.3442 | 0.3809 | 0.4124 | 0.4488 | 0.5094 | 0.5770 | 0.6446 | 0.7053 | 0.7416 | 0.7732 | 0.8098 |
| 46 | 0.3471 | 0.3843 | 0.4162 | 0.4531 | 0.5146 | 0.5831 | 0.6516 | 0.7131 | 0.7499 | 0.7819 | 0.8191 |
| 47 | 0.3501 | 0.3877 | 0.4201 | 0.4575 | 0.5198 | 0.5893 | 0.6587 | 0.7211 | 0.7584 | 0.7908 | 0.8285 |
| 48 | 0.3531 | 0.3913 | 0.4241 | 0.4620 | 0.5252 | 0.5956 | 0.6660 | 0.7292 | 0.7671 | 0.7999 | 0.8381 |
| 49 | 0.3561 | 0.3948 | 0.4282 | 0.4666 | 0.5306 | 0.6020 | 0.6735 | 0.7375 | 0.7759 | 0.8092 | 0.8480 |
| 50 | 0.3592 | 0.3985 | 0.4323 | 0.4712 | 0.5362 | 0.6086 | 0.6810 | 0.7460 | 0.7850 | 0.8187 | 0.8580 |
| 51 | 0.3624 | 0.4023 | 0.4365 | 0.4760 | 0.5419 | 0.6153 | 0.6888 | 0.7547 | 0.7942 | 0.8284 | 0.8683 |
| 52 | 0.3657 | 0.4061 | 0.4408 | 0.4809 | 0.5477 | 0.6222 | 0.6967 | 0.7635 | 0.8036 | 0.8383 | 0.8787 |
| 53 | 0.3690 | 0.4100 | 0.4452 | 0.4859 | 0.5536 | 0.6292 | 0.7047 | 0.7725 | 0.8131 | 0.8484 | 0.8894 |
| 54 | 0.3724 | 0.4139 | 0.4497 | 0.4909 | 0.5597 | 0.6363 | 0.7129 | 0.7817 | 0.8229 | 0.8587 | 0.9002 |
| 55 | 0.3758 | 0.4180 | 0.4543 | 0.4961 | 0.5658 | 0.6436 | 0.7213 | 0.7910 | 0.8329 | 0.8691 | 0.9113 |
| 56 | 0.3793 | 0.4221 | 0.4589 | 0.5013 | 0.5721 | 0.6509 | 0.7298 | 0.8006 | 0.8430 | 0.8798 | 0.9225 |
| 57 | 0.3829 | 0.4263 | 0.4636 | 0.5067 | 0.5784 | 0.6585 | 0.7385 | 0.8103 | 0.8533 | 0.8906 | 0.9340 |
| 58 | 0.3865 | 0.4306 | 0.4684 | 0.5121 | 0.5849 | 0.6661 | 0.7473 | 0.8201 | 0.8638 | 0.9016 | 0.9457 |
| 59 | 0.3902 | 0.4349 | 0.4733 | 0.5176 | 0.5915 | 0.6739 | 0.7562 | 0.8301 | 0.8744 | 0.9129 | 0.9575 |
| 60 | 0.3940 | 0.4393 | 0.4783 | 0.5232 | 0.5982 | 0.6818 | 0.7654 | 0.8404 | 0.8853 | 0.9243 | 0.9696 |
| 61 | 0.3978 | 0.4438 | 0.4834 | 0.5290 | 0.6050 | 0.6898 | 0.7746 | 0.8507 | 0.8963 | 0.9359 | 0.9819 |
| 62 | 0.4017 | 0.4484 | 0.4885 | 0.5348 | 0.6120 | 0.6980 | 0.7841 | 0.8613 | 0.9076 | 0.9477 | 0.9944 |
| 63 | 0.4056 | 0.4530 | 0.4937 | 0.5407 | 0.6190 | 0.7063 | 0.7937 | 0.8720 | 0.9190 | 0.9597 | 1.0071 |
| 64 | 0.4096 | 0.4577 | 0.4990 | 0.5467 | 0.6262 | 0.7148 | 0.8034 | 0.8829 | 0.9305 | 0.9719 | 1.0199 |
| 65 | 0.4137 | 0.4625 | 0.5044 | 0.5528 | 0.6335 | 0.7234 | 0.8133 | 0.8940 | 0.9423 | 0.9843 | 1.0330 |
| 66 | 0.4179 | 0.4673 | 0.5099 | 0.5590 | 0.6408 | 0.7321 | 0.8233 | 0.9052 | 0.9543 | 0.9968 | 1.0463 |
| 67 | 0.4221 | 0.4723 | 0.5155 | 0.5653 | 0.6483 | 0.7409 | 0.8335 | 0.9166 | 0.9664 | 1.0096 | 1.0598 |
| 68 | 0.4263 | 0.4773 | 0.5211 | 0.5717 | 0.6560 | 0.7499 | 0.8439 | 0.9282 | 0.9787 | 1.0225 | 1.0735 |
| 69 | 0.4307 | 0.4824 | 0.5268 | 0.5781 | 0.6637 | 0.7590 | 0.8544 | 0.9399 | 0.9912 | 1.0357 | 1.0874 |
| 70 | 0.4350 | 0.4875 | 0.5327 | 0.5847 | 0.6715 | 0.7683 | 0.8650 | 0.9518 | 1.0039 | 1.0490 | 1.1015 |
| 71 | 0.4395 | 0.4928 | 0.5386 | 0.5914 | 0.6795 | 0.7777 | 0.8758 | 0.9639 | 1.0167 | 1.0625 | 1.1158 |
| 72 | 0.4440 | 0.4981 | 0.5445 | 0.5981 | 0.6875 | 0.7872 | 0.8868 | 0.9762 | 1.0298 | 1.0763 | 1.1303 |
| 73 | 0.4486 | 0.5034 | 0.5506 | 0.6050 | 0.6957 | 0.7968 | 0.8979 | 0.9886 | 1.0430 | 1.0902 | 1.1450 |

| **Table D. Right CIMT [mm] percentiles for FEMALE healthy subjects** | | | | | | | | | | | |
| --- | --- | --- | --- | --- | --- | --- | --- | --- | --- | --- | --- |
|  |  |  |  |  |  |  |  |  |  |  |  |
| **Age [years]** | **1 th** | **2.5 th** | **5 th** | **10 th** | **25 th** | **50 th** | **75 th** | **90 th** | **95 th** | **97.5 th** | **99 th** |
| 23 | 0.3150 | 0.3360 | 0.3540 | 0.3748 | 0.4095 | 0.4482 | 0.4869 | 0.5216 | 0.5424 | 0.5604 | 0.5814 |
| 24 | 0.3167 | 0.3380 | 0.3564 | 0.3775 | 0.4128 | 0.4521 | 0.4914 | 0.5267 | 0.5479 | 0.5662 | 0.5876 |
| 25 | 0.3184 | 0.3401 | 0.3588 | 0.3803 | 0.4162 | 0.4562 | 0.4962 | 0.5321 | 0.5536 | 0.5723 | 0.5940 |
| 26 | 0.3203 | 0.3423 | 0.3613 | 0.3832 | 0.4198 | 0.4605 | 0.5012 | 0.5377 | 0.5596 | 0.5786 | 0.6007 |
| 27 | 0.3221 | 0.3446 | 0.3640 | 0.3863 | 0.4235 | 0.4649 | 0.5064 | 0.5436 | 0.5659 | 0.5852 | 0.6077 |
| 28 | 0.3241 | 0.3470 | 0.3667 | 0.3894 | 0.4273 | 0.4695 | 0.5117 | 0.5496 | 0.5723 | 0.5920 | 0.6149 |
| 29 | 0.3261 | 0.3495 | 0.3695 | 0.3927 | 0.4313 | 0.4743 | 0.5173 | 0.5559 | 0.5790 | 0.5991 | 0.6224 |
| 30 | 0.3282 | 0.3520 | 0.3725 | 0.3960 | 0.4354 | 0.4792 | 0.5231 | 0.5624 | 0.5860 | 0.6064 | 0.6302 |
| 31 | 0.3304 | 0.3546 | 0.3755 | 0.3995 | 0.4396 | 0.4843 | 0.5290 | 0.5691 | 0.5931 | 0.6140 | 0.6382 |
| 32 | 0.3327 | 0.3574 | 0.3786 | 0.4031 | 0.4440 | 0.4896 | 0.5352 | 0.5760 | 0.6005 | 0.6218 | 0.6465 |
| 33 | 0.3350 | 0.3602 | 0.3819 | 0.4069 | 0.4485 | 0.4950 | 0.5415 | 0.5832 | 0.6082 | 0.6299 | 0.6551 |
| 34 | 0.3374 | 0.3631 | 0.3852 | 0.4107 | 0.4532 | 0.5006 | 0.5480 | 0.5906 | 0.6161 | 0.6382 | 0.6639 |
| 35 | 0.3398 | 0.3661 | 0.3886 | 0.4146 | 0.4580 | 0.5064 | 0.5548 | 0.5982 | 0.6242 | 0.6467 | 0.6730 |
| 36 | 0.3423 | 0.3691 | 0.3921 | 0.4187 | 0.4630 | 0.5123 | 0.5617 | 0.6060 | 0.6325 | 0.6556 | 0.6823 |
| 37 | 0.3449 | 0.3723 | 0.3958 | 0.4229 | 0.4681 | 0.5184 | 0.5688 | 0.6140 | 0.6411 | 0.6646 | 0.6919 |
| 38 | 0.3476 | 0.3755 | 0.3995 | 0.4272 | 0.4733 | 0.5247 | 0.5761 | 0.6223 | 0.6499 | 0.6739 | 0.7018 |
| 39 | 0.3504 | 0.3788 | 0.4033 | 0.4316 | 0.4787 | 0.5312 | 0.5836 | 0.6307 | 0.6590 | 0.6835 | 0.7119 |
| 40 | 0.3532 | 0.3823 | 0.4073 | 0.4361 | 0.4842 | 0.5378 | 0.5914 | 0.6394 | 0.6683 | 0.6933 | 0.7223 |
| 41 | 0.3561 | 0.3857 | 0.4113 | 0.4407 | 0.4898 | 0.5445 | 0.5993 | 0.6484 | 0.6778 | 0.7033 | 0.7330 |
| 42 | 0.3590 | 0.3893 | 0.4154 | 0.4455 | 0.4956 | 0.5515 | 0.6074 | 0.6575 | 0.6875 | 0.7136 | 0.7439 |
| 43 | 0.3621 | 0.3930 | 0.4196 | 0.4503 | 0.5015 | 0.5586 | 0.6157 | 0.6669 | 0.6975 | 0.7242 | 0.7551 |
| 44 | 0.3652 | 0.3968 | 0.4240 | 0.4553 | 0.5076 | 0.5659 | 0.6241 | 0.6764 | 0.7078 | 0.7350 | 0.7666 |
| 45 | 0.3683 | 0.4006 | 0.4284 | 0.4604 | 0.5138 | 0.5733 | 0.6328 | 0.6862 | 0.7182 | 0.7460 | 0.7783 |
| 46 | 0.3716 | 0.4045 | 0.4329 | 0.4656 | 0.5201 | 0.5809 | 0.6417 | 0.6962 | 0.7289 | 0.7573 | 0.7903 |
| 47 | 0.3749 | 0.4086 | 0.4375 | 0.4709 | 0.5266 | 0.5887 | 0.6508 | 0.7065 | 0.7399 | 0.7688 | 0.8025 |
| 48 | 0.3783 | 0.4127 | 0.4422 | 0.4763 | 0.5332 | 0.5966 | 0.6600 | 0.7169 | 0.7510 | 0.7806 | 0.8150 |
| 49 | 0.3817 | 0.4169 | 0.4471 | 0.4819 | 0.5400 | 0.6047 | 0.6695 | 0.7276 | 0.7624 | 0.7926 | 0.8278 |
| 50 | 0.3853 | 0.4211 | 0.4520 | 0.4876 | 0.5469 | 0.6130 | 0.6792 | 0.7385 | 0.7741 | 0.8049 | 0.8408 |
| 51 | 0.3889 | 0.4255 | 0.4570 | 0.4933 | 0.5539 | 0.6215 | 0.6890 | 0.7496 | 0.7860 | 0.8175 | 0.8541 |
| 52 | 0.3925 | 0.4299 | 0.4621 | 0.4992 | 0.5611 | 0.6301 | 0.6991 | 0.7610 | 0.7981 | 0.8302 | 0.8677 |
| 53 | 0.3963 | 0.4345 | 0.4673 | 0.5052 | 0.5684 | 0.6389 | 0.7093 | 0.7725 | 0.8104 | 0.8433 | 0.8815 |
| 54 | 0.4001 | 0.4391 | 0.4727 | 0.5113 | 0.5759 | 0.6478 | 0.7198 | 0.7843 | 0.8230 | 0.8565 | 0.8956 |
| 55 | 0.4040 | 0.4438 | 0.4781 | 0.5176 | 0.5835 | 0.6569 | 0.7304 | 0.7963 | 0.8358 | 0.8701 | 0.9099 |
| 56 | 0.4079 | 0.4486 | 0.4836 | 0.5239 | 0.5912 | 0.6662 | 0.7412 | 0.8085 | 0.8489 | 0.8838 | 0.9245 |
| 57 | 0.4119 | 0.4535 | 0.4892 | 0.5304 | 0.5991 | 0.6757 | 0.7522 | 0.8210 | 0.8621 | 0.8979 | 0.9394 |
| 58 | 0.4160 | 0.4584 | 0.4949 | 0.5370 | 0.6071 | 0.6853 | 0.7635 | 0.8336 | 0.8757 | 0.9121 | 0.9545 |
| 59 | 0.4202 | 0.4635 | 0.5007 | 0.5437 | 0.6153 | 0.6951 | 0.7749 | 0.8465 | 0.8894 | 0.9266 | 0.9699 |
| 60 | 0.4245 | 0.4686 | 0.5066 | 0.5505 | 0.6236 | 0.7050 | 0.7865 | 0.8596 | 0.9034 | 0.9414 | 0.9856 |
| 61 | 0.4288 | 0.4739 | 0.5127 | 0.5574 | 0.6320 | 0.7151 | 0.7983 | 0.8729 | 0.9176 | 0.9564 | 1.0015 |
| 62 | 0.4332 | 0.4792 | 0.5188 | 0.5644 | 0.6406 | 0.7254 | 0.8103 | 0.8864 | 0.9321 | 0.9717 | 1.0177 |
| 63 | 0.4376 | 0.4846 | 0.5250 | 0.5716 | 0.6493 | 0.7359 | 0.8225 | 0.9002 | 0.9468 | 0.9872 | 1.0342 |
| 64 | 0.4421 | 0.4901 | 0.5313 | 0.5788 | 0.6581 | 0.7465 | 0.8349 | 0.9142 | 0.9617 | 1.0029 | 1.0509 |
| 65 | 0.4467 | 0.4956 | 0.5377 | 0.5862 | 0.6671 | 0.7573 | 0.8475 | 0.9284 | 0.9769 | 1.0190 | 1.0679 |
| 66 | 0.4514 | 0.5013 | 0.5442 | 0.5937 | 0.6762 | 0.7683 | 0.8603 | 0.9428 | 0.9923 | 1.0352 | 1.0851 |
| 67 | 0.4561 | 0.5070 | 0.5508 | 0.6013 | 0.6855 | 0.7794 | 0.8732 | 0.9574 | 1.0079 | 1.0517 | 1.1026 |
| 68 | 0.4610 | 0.5129 | 0.5575 | 0.6090 | 0.6949 | 0.7907 | 0.8864 | 0.9723 | 1.0238 | 1.0685 | 1.1204 |
| 69 | 0.4658 | 0.5188 | 0.5643 | 0.6169 | 0.7045 | 0.8021 | 0.8998 | 0.9874 | 1.0399 | 1.0855 | 1.1384 |
| 70 | 0.4708 | 0.5248 | 0.5713 | 0.6248 | 0.7142 | 0.8137 | 0.9133 | 1.0027 | 1.0562 | 1.1027 | 1.1567 |
| 71 | 0.4758 | 0.5309 | 0.5783 | 0.6329 | 0.7240 | 0.8255 | 0.9271 | 1.0182 | 1.0728 | 1.1202 | 1.1753 |
| 72 | 0.4809 | 0.5371 | 0.5854 | 0.6411 | 0.7340 | 0.8375 | 0.9410 | 1.0339 | 1.0896 | 1.1379 | 1.1941 |
| 73 | 0.4861 | 0.5433 | 0.5926 | 0.6494 | 0.7441 | 0.8496 | 0.9552 | 1.0499 | 1.1067 | 1.1559 | 1.2132 |
| 74 | 0.4913 | 0.5497 | 0.5999 | 0.6578 | 0.7543 | 0.8619 | 0.9695 | 1.0661 | 1.1240 | 1.1742 | 1.2325 |
| 75 | 0.4966 | 0.5561 | 0.6073 | 0.6663 | 0.7647 | 0.8744 | 0.9841 | 1.0825 | 1.1415 | 1.1926 | 1.2521 |
| 76 | 0.5020 | 0.5626 | 0.6148 | 0.6749 | 0.7752 | 0.8870 | 0.9988 | 1.0991 | 1.1592 | 1.2114 | 1.2720 |
| 77 | 0.5075 | 0.5693 | 0.6224 | 0.6837 | 0.7859 | 0.8998 | 1.0137 | 1.1159 | 1.1772 | 1.2304 | 1.2922 |
| 78 | 0.5130 | 0.5759 | 0.6301 | 0.6925 | 0.7967 | 0.9128 | 1.0289 | 1.1330 | 1.1954 | 1.2496 | 1.3126 |

| **Table E. Left CIMT [mm] percentiles for MALE healthy subjects** | | | | | | | | | | | |
| --- | --- | --- | --- | --- | --- | --- | --- | --- | --- | --- | --- |
|  |  |  |  |  |  |  |  |  |  |  |  |
| **Age [years]** | **1 th** | **2.5 th** | **5 th** | **10 th** | **25 th** | **50 th** | **75 th** | **90 th** | **95 th** | **97.5 th** | **99 th** |
| 20 | 0.3292 | 0.3533 | 0.3740 | 0.3979 | 0.4378 | 0.4822 | 0.5267 | 0.5665 | 0.5904 | 0.6112 | 0.6353 |
| 21 | 0.3301 | 0.3545 | 0.3756 | 0.3998 | 0.4403 | 0.4854 | 0.5306 | 0.5710 | 0.5953 | 0.6163 | 0.6408 |
| 22 | 0.3310 | 0.3558 | 0.3772 | 0.4019 | 0.4430 | 0.4888 | 0.5346 | 0.5758 | 0.6004 | 0.6218 | 0.6466 |
| 23 | 0.3320 | 0.3572 | 0.3789 | 0.4040 | 0.4458 | 0.4923 | 0.5389 | 0.5807 | 0.6057 | 0.6275 | 0.6527 |
| 24 | 0.3330 | 0.3587 | 0.3807 | 0.4062 | 0.4487 | 0.4960 | 0.5434 | 0.5859 | 0.6113 | 0.6334 | 0.6591 |
| 25 | 0.3340 | 0.3601 | 0.3826 | 0.4085 | 0.4517 | 0.4999 | 0.5480 | 0.5912 | 0.6171 | 0.6396 | 0.6657 |
| 26 | 0.3351 | 0.3617 | 0.3846 | 0.4109 | 0.4549 | 0.5039 | 0.5529 | 0.5969 | 0.6232 | 0.6461 | 0.6726 |
| 27 | 0.3363 | 0.3633 | 0.3866 | 0.4134 | 0.4582 | 0.5080 | 0.5579 | 0.6027 | 0.6295 | 0.6528 | 0.6798 |
| 28 | 0.3375 | 0.3650 | 0.3887 | 0.4160 | 0.4616 | 0.5124 | 0.5631 | 0.6087 | 0.6360 | 0.6597 | 0.6873 |
| 29 | 0.3387 | 0.3667 | 0.3909 | 0.4187 | 0.4651 | 0.5168 | 0.5686 | 0.6150 | 0.6428 | 0.6669 | 0.6950 |
| 30 | 0.3400 | 0.3685 | 0.3931 | 0.4215 | 0.4688 | 0.5215 | 0.5742 | 0.6215 | 0.6498 | 0.6744 | 0.7030 |
| 31 | 0.3413 | 0.3704 | 0.3955 | 0.4244 | 0.4725 | 0.5263 | 0.5800 | 0.6282 | 0.6570 | 0.6821 | 0.7112 |
| 32 | 0.3426 | 0.3723 | 0.3979 | 0.4273 | 0.4765 | 0.5312 | 0.5860 | 0.6351 | 0.6645 | 0.6901 | 0.7198 |
| 33 | 0.3440 | 0.3743 | 0.4004 | 0.4304 | 0.4805 | 0.5363 | 0.5921 | 0.6422 | 0.6722 | 0.6983 | 0.7286 |
| 34 | 0.3455 | 0.3764 | 0.4029 | 0.4336 | 0.4846 | 0.5416 | 0.5985 | 0.6496 | 0.6802 | 0.7068 | 0.7376 |
| 35 | 0.3470 | 0.3785 | 0.4056 | 0.4368 | 0.4889 | 0.5470 | 0.6051 | 0.6572 | 0.6884 | 0.7155 | 0.7470 |
| 36 | 0.3485 | 0.3806 | 0.4083 | 0.4402 | 0.4933 | 0.5526 | 0.6118 | 0.6650 | 0.6968 | 0.7245 | 0.7566 |
| 37 | 0.3501 | 0.3829 | 0.4111 | 0.4436 | 0.4978 | 0.5583 | 0.6187 | 0.6730 | 0.7055 | 0.7337 | 0.7665 |
| 38 | 0.3517 | 0.3852 | 0.4139 | 0.4471 | 0.5025 | 0.5642 | 0.6259 | 0.6812 | 0.7144 | 0.7432 | 0.7766 |
| 39 | 0.3534 | 0.3875 | 0.4169 | 0.4508 | 0.5073 | 0.5702 | 0.6332 | 0.6897 | 0.7235 | 0.7529 | 0.7871 |
| 40 | 0.3551 | 0.3899 | 0.4199 | 0.4545 | 0.5122 | 0.5764 | 0.6407 | 0.6984 | 0.7329 | 0.7629 | 0.7978 |
| 41 | 0.3568 | 0.3924 | 0.4230 | 0.4583 | 0.5172 | 0.5828 | 0.6484 | 0.7073 | 0.7425 | 0.7732 | 0.8087 |
| 42 | 0.3586 | 0.3949 | 0.4262 | 0.4622 | 0.5223 | 0.5893 | 0.6563 | 0.7164 | 0.7524 | 0.7837 | 0.8200 |
| 43 | 0.3604 | 0.3975 | 0.4294 | 0.4662 | 0.5276 | 0.5960 | 0.6644 | 0.7257 | 0.7625 | 0.7944 | 0.8315 |
| 44 | 0.3623 | 0.4002 | 0.4328 | 0.4703 | 0.5330 | 0.6028 | 0.6726 | 0.7353 | 0.7728 | 0.8054 | 0.8433 |
| 45 | 0.3642 | 0.4029 | 0.4362 | 0.4745 | 0.5385 | 0.6098 | 0.6811 | 0.7451 | 0.7834 | 0.8167 | 0.8553 |
| 46 | 0.3662 | 0.4057 | 0.4397 | 0.4788 | 0.5441 | 0.6169 | 0.6897 | 0.7550 | 0.7942 | 0.8282 | 0.8677 |
| 47 | 0.3682 | 0.4085 | 0.4432 | 0.4832 | 0.5499 | 0.6242 | 0.6986 | 0.7653 | 0.8052 | 0.8399 | 0.8802 |
| 48 | 0.3703 | 0.4114 | 0.4469 | 0.4877 | 0.5558 | 0.6317 | 0.7076 | 0.7757 | 0.8165 | 0.8519 | 0.8931 |
| 49 | 0.3724 | 0.4144 | 0.4506 | 0.4923 | 0.5618 | 0.6393 | 0.7168 | 0.7864 | 0.8280 | 0.8642 | 0.9062 |
| 50 | 0.3745 | 0.4174 | 0.4544 | 0.4969 | 0.5679 | 0.6471 | 0.7262 | 0.7972 | 0.8398 | 0.8767 | 0.9197 |
| 51 | 0.3767 | 0.4205 | 0.4582 | 0.5017 | 0.5742 | 0.6550 | 0.7358 | 0.8083 | 0.8518 | 0.8895 | 0.9333 |
| 52 | 0.3789 | 0.4237 | 0.4622 | 0.5065 | 0.5806 | 0.6631 | 0.7456 | 0.8196 | 0.8640 | 0.9025 | 0.9473 |
| 53 | 0.3812 | 0.4269 | 0.4662 | 0.5115 | 0.5871 | 0.6713 | 0.7556 | 0.8312 | 0.8765 | 0.9158 | 0.9615 |
| 54 | 0.3835 | 0.4301 | 0.4703 | 0.5165 | 0.5937 | 0.6797 | 0.7658 | 0.8429 | 0.8892 | 0.9293 | 0.9760 |
| 55 | 0.3858 | 0.4335 | 0.4744 | 0.5217 | 0.6005 | 0.6883 | 0.7761 | 0.8549 | 0.9021 | 0.9431 | 0.9908 |
| 56 | 0.3882 | 0.4369 | 0.4787 | 0.5269 | 0.6073 | 0.6970 | 0.7867 | 0.8671 | 0.9153 | 0.9572 | 1.0058 |
| 57 | 0.3907 | 0.4403 | 0.4830 | 0.5322 | 0.6144 | 0.7059 | 0.7974 | 0.8795 | 0.9287 | 0.9714 | 1.0211 |
| 58 | 0.3932 | 0.4438 | 0.4874 | 0.5377 | 0.6215 | 0.7149 | 0.8083 | 0.8922 | 0.9424 | 0.9860 | 1.0367 |
| 59 | 0.3957 | 0.4474 | 0.4919 | 0.5432 | 0.6287 | 0.7241 | 0.8195 | 0.9050 | 0.9563 | 1.0008 | 1.0525 |
| 60 | 0.3982 | 0.4510 | 0.4964 | 0.5488 | 0.6361 | 0.7334 | 0.8308 | 0.9181 | 0.9704 | 1.0158 | 1.0686 |
| 61 | 0.4009 | 0.4547 | 0.5011 | 0.5545 | 0.6436 | 0.7429 | 0.8423 | 0.9314 | 0.9848 | 1.0311 | 1.0850 |
| 62 | 0.4035 | 0.4585 | 0.5058 | 0.5603 | 0.6512 | 0.7526 | 0.8539 | 0.9449 | 0.9994 | 1.0467 | 1.1017 |
| 63 | 0.4062 | 0.4623 | 0.5106 | 0.5662 | 0.6590 | 0.7624 | 0.8658 | 0.9586 | 1.0142 | 1.0625 | 1.1186 |
| 64 | 0.4090 | 0.4662 | 0.5154 | 0.5722 | 0.6668 | 0.7724 | 0.8779 | 0.9726 | 1.0293 | 1.0786 | 1.1358 |
| 65 | 0.4117 | 0.4701 | 0.5204 | 0.5783 | 0.6748 | 0.7825 | 0.8902 | 0.9867 | 1.0446 | 1.0949 | 1.1533 |
| 66 | 0.4146 | 0.4741 | 0.5254 | 0.5844 | 0.6830 | 0.7928 | 0.9026 | 1.0011 | 1.0602 | 1.1114 | 1.1710 |
| 67 | 0.4174 | 0.4782 | 0.5305 | 0.5907 | 0.6912 | 0.8032 | 0.9152 | 1.0157 | 1.0760 | 1.1283 | 1.1890 |
| 68 | 0.4204 | 0.4823 | 0.5356 | 0.5971 | 0.6996 | 0.8138 | 0.9281 | 1.0306 | 1.0920 | 1.1453 | 1.2073 |
| 69 | 0.4233 | 0.4865 | 0.5409 | 0.6035 | 0.7081 | 0.8246 | 0.9411 | 1.0456 | 1.1083 | 1.1626 | 1.2258 |
| 70 | 0.4263 | 0.4908 | 0.5462 | 0.6101 | 0.7167 | 0.8355 | 0.9543 | 1.0609 | 1.1248 | 1.1802 | 1.2447 |
| 71 | 0.4294 | 0.4951 | 0.5516 | 0.6167 | 0.7254 | 0.8466 | 0.9677 | 1.0764 | 1.1415 | 1.1981 | 1.2638 |
| 72 | 0.4324 | 0.4994 | 0.5570 | 0.6235 | 0.7343 | 0.8578 | 0.9813 | 1.0921 | 1.1585 | 1.2161 | 1.2831 |
| 73 | 0.4356 | 0.5039 | 0.5626 | 0.6303 | 0.7433 | 0.8692 | 0.9951 | 1.1080 | 1.1757 | 1.2345 | 1.3028 |

| **Table F. Left CIMT [mm] percentiles for FEMALE healthy subjects** | | | | | | | | | | | |
| --- | --- | --- | --- | --- | --- | --- | --- | --- | --- | --- | --- |
|  |  |  |  |  |  |  |  |  |  |  |  |
| **Age [years]** | **1 th** | **2.5 th** | **5 th** | **10 th** | **25 th** | **50 th** | **75 th** | **90 th** | **95 th** | **97.5 th** | **99 th** |
| 23 | 0.3021 | 0.3260 | 0.3466 | 0.3704 | 0.4100 | 0.4542 | 0.4984 | 0.5380 | 0.5618 | 0.5824 | 0.6064 |
| 24 | 0.3041 | 0.3285 | 0.3494 | 0.3735 | 0.4138 | 0.4586 | 0.5035 | 0.5437 | 0.5679 | 0.5888 | 0.6131 |
| 25 | 0.3063 | 0.3310 | 0.3523 | 0.3768 | 0.4177 | 0.4632 | 0.5088 | 0.5496 | 0.5741 | 0.5954 | 0.6201 |
| 26 | 0.3086 | 0.3337 | 0.3553 | 0.3802 | 0.4217 | 0.4680 | 0.5143 | 0.5558 | 0.5807 | 0.6023 | 0.6274 |
| 27 | 0.3110 | 0.3365 | 0.3584 | 0.3837 | 0.4259 | 0.4730 | 0.5200 | 0.5622 | 0.5875 | 0.6095 | 0.6350 |
| 28 | 0.3134 | 0.3394 | 0.3617 | 0.3874 | 0.4303 | 0.4781 | 0.5259 | 0.5689 | 0.5946 | 0.6169 | 0.6428 |
| 29 | 0.3160 | 0.3423 | 0.3650 | 0.3912 | 0.4348 | 0.4835 | 0.5321 | 0.5757 | 0.6019 | 0.6246 | 0.6510 |
| 30 | 0.3186 | 0.3454 | 0.3685 | 0.3951 | 0.4395 | 0.4890 | 0.5385 | 0.5829 | 0.6095 | 0.6326 | 0.6594 |
| 31 | 0.3213 | 0.3486 | 0.3721 | 0.3992 | 0.4444 | 0.4947 | 0.5451 | 0.5902 | 0.6173 | 0.6408 | 0.6681 |
| 32 | 0.3241 | 0.3519 | 0.3758 | 0.4034 | 0.4494 | 0.5006 | 0.5519 | 0.5978 | 0.6254 | 0.6493 | 0.6771 |
| 33 | 0.3270 | 0.3553 | 0.3796 | 0.4077 | 0.4545 | 0.5067 | 0.5589 | 0.6057 | 0.6338 | 0.6581 | 0.6864 |
| 34 | 0.3300 | 0.3588 | 0.3836 | 0.4122 | 0.4598 | 0.5130 | 0.5661 | 0.6138 | 0.6424 | 0.6671 | 0.6960 |
| 35 | 0.3331 | 0.3624 | 0.3877 | 0.4168 | 0.4653 | 0.5194 | 0.5736 | 0.6221 | 0.6512 | 0.6765 | 0.7058 |
| 36 | 0.3362 | 0.3661 | 0.3918 | 0.4215 | 0.4710 | 0.5261 | 0.5812 | 0.6307 | 0.6603 | 0.6861 | 0.7160 |
| 37 | 0.3395 | 0.3699 | 0.3961 | 0.4264 | 0.4768 | 0.5329 | 0.5891 | 0.6395 | 0.6697 | 0.6959 | 0.7264 |
| 38 | 0.3428 | 0.3739 | 0.4006 | 0.4314 | 0.4827 | 0.5400 | 0.5972 | 0.6486 | 0.6794 | 0.7061 | 0.7371 |
| 39 | 0.3462 | 0.3779 | 0.4051 | 0.4365 | 0.4888 | 0.5472 | 0.6055 | 0.6579 | 0.6892 | 0.7165 | 0.7481 |
| 40 | 0.3498 | 0.3820 | 0.4098 | 0.4418 | 0.4951 | 0.5546 | 0.6141 | 0.6674 | 0.6994 | 0.7271 | 0.7594 |
| 41 | 0.3534 | 0.3863 | 0.4145 | 0.4472 | 0.5015 | 0.5622 | 0.6228 | 0.6772 | 0.7098 | 0.7381 | 0.7710 |
| 42 | 0.3571 | 0.3906 | 0.4194 | 0.4527 | 0.5081 | 0.5700 | 0.6318 | 0.6872 | 0.7205 | 0.7493 | 0.7828 |
| 43 | 0.3609 | 0.3950 | 0.4244 | 0.4583 | 0.5149 | 0.5779 | 0.6409 | 0.6975 | 0.7314 | 0.7608 | 0.7950 |
| 44 | 0.3647 | 0.3996 | 0.4296 | 0.4641 | 0.5218 | 0.5861 | 0.6503 | 0.7080 | 0.7426 | 0.7725 | 0.8074 |
| 45 | 0.3687 | 0.4043 | 0.4348 | 0.4701 | 0.5289 | 0.5944 | 0.6599 | 0.7187 | 0.7540 | 0.7846 | 0.8201 |
| 46 | 0.3728 | 0.4090 | 0.4402 | 0.4761 | 0.5361 | 0.6029 | 0.6698 | 0.7297 | 0.7657 | 0.7969 | 0.8331 |
| 47 | 0.3769 | 0.4139 | 0.4457 | 0.4823 | 0.5435 | 0.6117 | 0.6798 | 0.7410 | 0.7776 | 0.8094 | 0.8464 |
| 48 | 0.3811 | 0.4188 | 0.4513 | 0.4887 | 0.5510 | 0.6206 | 0.6901 | 0.7524 | 0.7898 | 0.8223 | 0.8600 |
| 49 | 0.3855 | 0.4239 | 0.4570 | 0.4951 | 0.5587 | 0.6296 | 0.7005 | 0.7642 | 0.8023 | 0.8354 | 0.8738 |
| 50 | 0.3899 | 0.4291 | 0.4628 | 0.5017 | 0.5666 | 0.6389 | 0.7112 | 0.7761 | 0.8150 | 0.8488 | 0.8880 |
| 51 | 0.3944 | 0.4344 | 0.4688 | 0.5084 | 0.5746 | 0.6484 | 0.7221 | 0.7883 | 0.8280 | 0.8624 | 0.9024 |
| 52 | 0.3990 | 0.4398 | 0.4749 | 0.5153 | 0.5828 | 0.6580 | 0.7333 | 0.8008 | 0.8412 | 0.8763 | 0.9171 |
| 53 | 0.4036 | 0.4452 | 0.4810 | 0.5223 | 0.5911 | 0.6679 | 0.7446 | 0.8134 | 0.8547 | 0.8905 | 0.9321 |
| 54 | 0.4084 | 0.4508 | 0.4873 | 0.5294 | 0.5996 | 0.6779 | 0.7562 | 0.8264 | 0.8685 | 0.9050 | 0.9474 |
| 55 | 0.4133 | 0.4565 | 0.4938 | 0.5367 | 0.6083 | 0.6881 | 0.7679 | 0.8395 | 0.8825 | 0.9197 | 0.9630 |
| 56 | 0.4182 | 0.4623 | 0.5003 | 0.5441 | 0.6171 | 0.6985 | 0.7799 | 0.8529 | 0.8967 | 0.9347 | 0.9788 |
| 57 | 0.4232 | 0.4683 | 0.5070 | 0.5516 | 0.6261 | 0.7091 | 0.7921 | 0.8666 | 0.9112 | 0.9500 | 0.9950 |
| 58 | 0.4284 | 0.4743 | 0.5138 | 0.5593 | 0.6352 | 0.7199 | 0.8045 | 0.8805 | 0.9260 | 0.9655 | 1.0114 |
| 59 | 0.4336 | 0.4804 | 0.5207 | 0.5671 | 0.6445 | 0.7308 | 0.8172 | 0.8946 | 0.9410 | 0.9813 | 1.0281 |
| 60 | 0.4389 | 0.4866 | 0.5277 | 0.5750 | 0.6540 | 0.7420 | 0.8300 | 0.9090 | 0.9563 | 0.9974 | 1.0451 |
| 61 | 0.4443 | 0.4929 | 0.5348 | 0.5831 | 0.6636 | 0.7533 | 0.8431 | 0.9236 | 0.9719 | 1.0137 | 1.0624 |
| 62 | 0.4497 | 0.4994 | 0.5421 | 0.5913 | 0.6734 | 0.7649 | 0.8564 | 0.9385 | 0.9877 | 1.0304 | 1.0800 |
| 63 | 0.4553 | 0.5059 | 0.5494 | 0.5996 | 0.6833 | 0.7766 | 0.8699 | 0.9536 | 1.0037 | 1.0472 | 1.0978 |
| 64 | 0.4610 | 0.5126 | 0.5569 | 0.6081 | 0.6934 | 0.7885 | 0.8836 | 0.9689 | 1.0200 | 1.0644 | 1.1160 |
| 65 | 0.4667 | 0.5193 | 0.5645 | 0.6167 | 0.7036 | 0.8006 | 0.8975 | 0.9845 | 1.0366 | 1.0818 | 1.1344 |
| 66 | 0.4726 | 0.5261 | 0.5722 | 0.6254 | 0.7140 | 0.8128 | 0.9117 | 1.0003 | 1.0534 | 1.0995 | 1.1531 |
| 67 | 0.4785 | 0.5331 | 0.5801 | 0.6342 | 0.7246 | 0.8253 | 0.9260 | 1.0164 | 1.0705 | 1.1175 | 1.1721 |
| 68 | 0.4845 | 0.5402 | 0.5880 | 0.6432 | 0.7353 | 0.8380 | 0.9406 | 1.0327 | 1.0879 | 1.1358 | 1.1914 |
| 69 | 0.4906 | 0.5473 | 0.5961 | 0.6524 | 0.7462 | 0.8508 | 0.9554 | 1.0492 | 1.1055 | 1.1543 | 1.2110 |
| 70 | 0.4968 | 0.5546 | 0.6043 | 0.6616 | 0.7572 | 0.8638 | 0.9704 | 1.0660 | 1.1233 | 1.1730 | 1.2308 |
| 71 | 0.5031 | 0.5620 | 0.6126 | 0.6710 | 0.7684 | 0.8770 | 0.9856 | 1.0830 | 1.1414 | 1.1921 | 1.2510 |
| 72 | 0.5094 | 0.5694 | 0.6211 | 0.6806 | 0.7798 | 0.8904 | 1.0011 | 1.1003 | 1.1598 | 1.2114 | 1.2714 |
| 73 | 0.5159 | 0.5770 | 0.6296 | 0.6902 | 0.7913 | 0.9040 | 1.0167 | 1.1178 | 1.1784 | 1.2310 | 1.2921 |
| 74 | 0.5225 | 0.5847 | 0.6383 | 0.7000 | 0.8030 | 0.9178 | 1.0326 | 1.1356 | 1.1973 | 1.2509 | 1.3131 |
| 75 | 0.5291 | 0.5925 | 0.6471 | 0.7099 | 0.8148 | 0.9318 | 1.0487 | 1.1536 | 1.2165 | 1.2710 | 1.3344 |
| 76 | 0.5358 | 0.6004 | 0.6560 | 0.7200 | 0.8268 | 0.9459 | 1.0650 | 1.1718 | 1.2359 | 1.2914 | 1.3560 |
| 77 | 0.5426 | 0.6084 | 0.6650 | 0.7302 | 0.8390 | 0.9602 | 1.0815 | 1.1903 | 1.2555 | 1.3121 | 1.3779 |
| 78 | 0.5495 | 0.6165 | 0.6741 | 0.7405 | 0.8513 | 0.9748 | 1.0982 | 1.2090 | 1.2754 | 1.3330 | 1.4000 |
|  |  |  |  |  |  |  |  |  |  |  |  |
